# The dynamics of Early Celtic consumption practices: A case study of the pottery from the Heuneburg

**DOI:** 10.1371/journal.pone.0222991

**Published:** 2019-10-23

**Authors:** Maxime Rageot, Angela Mötsch, Birgit Schorer, Andreas Gutekunst, Giulia Patrizi, Maximilian Zerrer, Sara Cafisso, Janine Fries-Knoblach, Leif Hansen, Roberto Tarpini, Dirk Krausse, Thomas Hoppe, Philipp W. Stockhammer, Cynthianne Spiteri

**Affiliations:** 1 Department of Pre- and Protohistory, University of Tübingen, Tübingen, Germany; 2 Institut für Vor- und Frühgeschichtliche Archäologie und Provinzialrömische Archäologie, Ludwig-Maximilians-Universität München, Munich, Germany; 3 Max Planck Institute for the Science of Human History, Jena, Germany; 4 Landesamt für Denkmalpflege im Regierungspräsidium Stuttgart, Esslingen, Germany; 5 Landesmuseum Württemberg, Stuttgart, Germany; University at Buffalo - The State University of New York, UNITED STATES

## Abstract

The Early Celtic site of the Heuneburg (Baden-Wuerttemberg, Germany) has long been understood as a hallmark of early urbanization in Central Europe. The rich collection of Mediterranean imports recovered from the settlement, the elite burials in its surroundings and the Mediterranean-inspired mudbrick fortification wall further point to the importance of intercultural connections with the Mediterranean as a crucial factor in the transformation of Early Iron Age societies. We describe a new facet of this process by studying the transformation of consumption practices, especially drinking habits, brought about by intercultural encounters from the late 7^th^ to the 5^th^ century BC through the analysis of organic remains in 133 ceramic vessels found at the Heuneburg using Organic Residue Analysis (ORA). During the Ha D1 phase, fermented beverages, including Mediterranean grape wine, were identified in and appear to have been consumed from local handmade ceramics. The latter were recovered from different status-related contexts within the Heuneburg, suggesting an early and well-established trade/exchange system of this Mediterranean product. This contrasts with the results obtained for the drinking and serving vessels from the Ha D3 phase that were studied. The consumption of fermented beverages (wine and especially bacteriofermented products) appears to have been concentrated on the plateau. The ORA analyses presented here seem to indicate that during this time, grape wine was consumed primarily from imported vessels, and more rarely from local prestigious fine wheel-made vessels. In addition to imported wine, we demonstrate the consumption of a wide variety of foodstuffs, such as animal fats (especially dairy products), millet, plant oils and waxy plants, fruit and beehive products as well as one or several other fermented beverage(s) that were probably locally produced. Through this diachronic study of vessel function from different intra-site contexts, we inform on changing and status-related practices of food processing and consumption.

## Introduction

The Early Celtic princely site of the “Heuneburg” is a key site for understanding Early Iron Age (7^th^–5^th^ centuries BC) societies in Central Europe. After early excavations of the princely burials around the Heuneburg in the 19^th^ century, comprehensive studies and excavations of the settlement on the Heuneburg plateau and its surroundings began in the 1950s and continue until today. This intensive fieldwork has brought to light an increasing number of imported Mediterranean objects, many of which were associated with feasting practices [1]. Scholars have long speculated whether these imports indicate the adoption of a Mediterranean lifestyle by the Early Celts [2].

The Heuneburg is considered a hallmark of the development of urbanization in the Early Iron Age north of the Alps [3, 4]. In the early 6^th^ century BC, the plateau of the Heuneburg was fortified with a mudbrick wall built following a Mediterranean construction typology, its lower settlement was adorned with a Mediterranean style gateway complex, and a large outer settlement was constructed [5]. During this period (Ha D1), the hilltop plateau was densely settled with rows of houses along a network of streets, whereas the large outer settlement was subdivided into an extensive system of different neighborhoods. The outer settlement appears to reveal the presence of farmstead-like compounds, some with evidence of artisanal production, within a larger agglomeration [6].

A massive conflagration largely destroyed the Heuneburg around 540/530 BC. Afterwards, most of the outer settlement was abandoned and neither the Mediterranean-style gateway nor the mudbrick wall were rebuilt. The latter was replaced by a wall constructed in a traditional method using earth and timber [5]. On the plateau, the relatively uniform row houses were replaced by more scattered buildings separated by fences and ditches [5]. At the same time, almost all imported Mediterranean pottery and locally wheel-made pottery made their first appearance.

**Fig 1:**
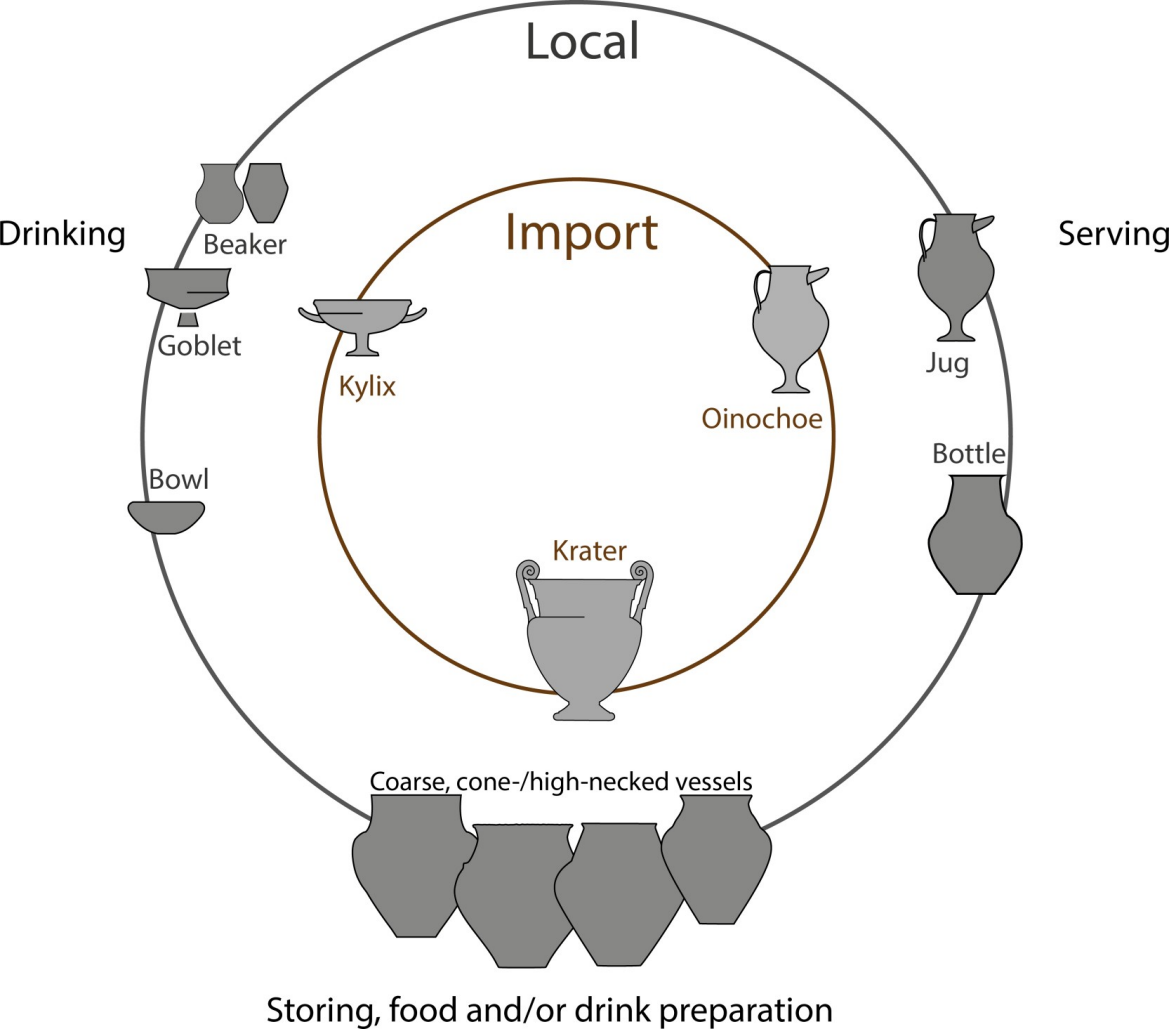
Selection of vessels shapes tested by ORA: Imported and local vessels of similar postulated function.

The complex spatial, social and economic setting of the Heuneburg and its transformation over time present the best basis for a better understanding of the dynamics of Early Celtic consumption practices. As part of this research, seven imported Attic ceramics and 126 local vessels from different contexts and different techno-typological categories classified according to their postulated functions were selected for ORA (Fig 1). These comprised:

- Drinking vessels including a Mediterranean kylix and local fine vessels (goblets, beakers, and bowls).
- Serving vessels whose shape suggests liquid or semi-liquid contents—these included two imported Attic oinochoe and local fine high forms (jugs, bottles).
- Vessels used for storage and the preparation of food and/or drink, including imported Attic kraters, local cone-necked vessels, local large and/or coarse vessels, bottles and bowls.

## Materials and methods

### Selection of archaeological samples

**Fig 2:**
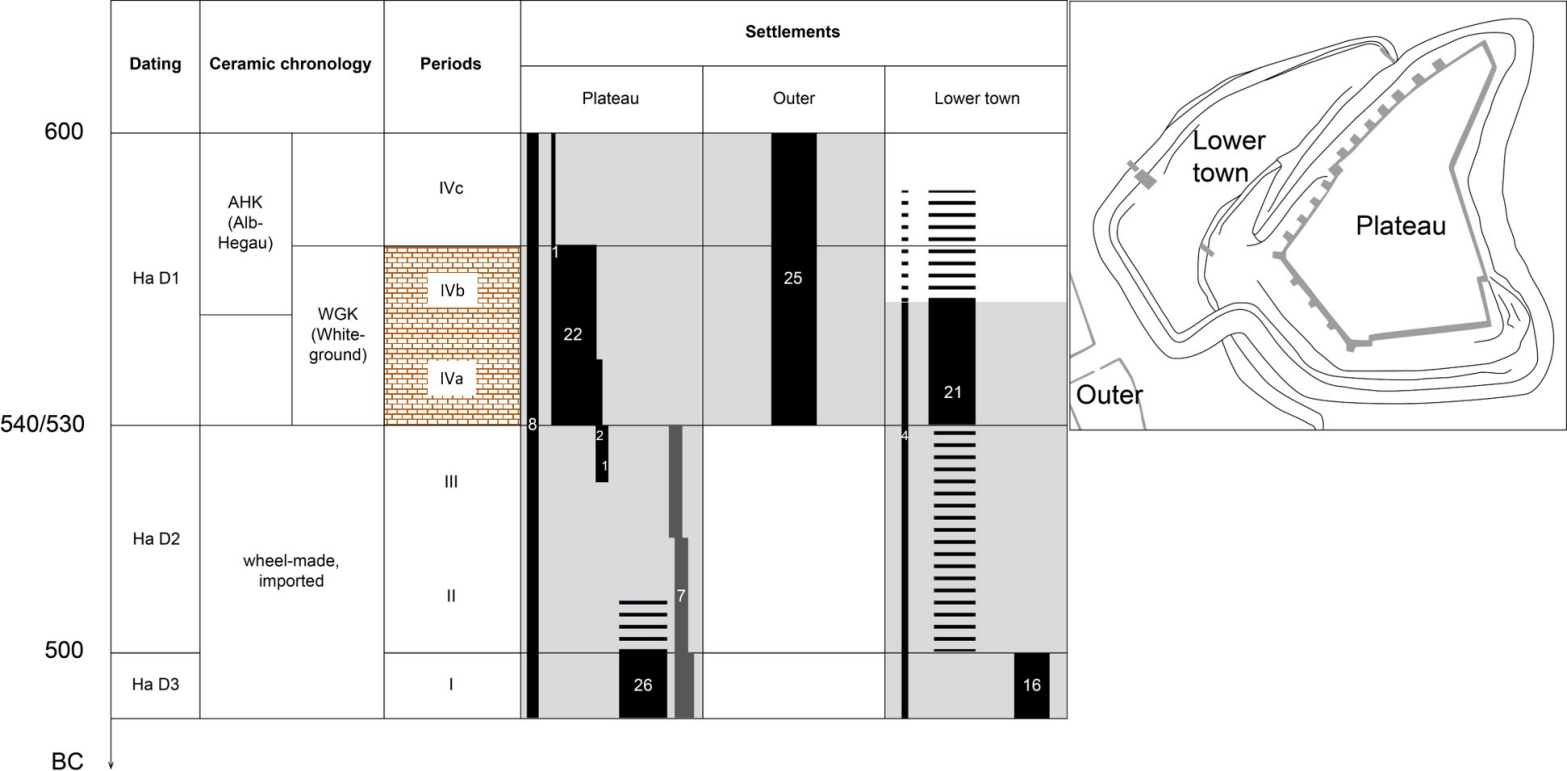
Archaeological contexts from which the selected vessels were recovered. Light grey filling: occupation phases of the Heuneburg settlements; black filling: contexts from which the selected local vessels were found; dark grey filling: contexts from which the selected imported vessels were recovered; red bricks: mudbrick wall periods (IVb and IVa). The map on the right corresponds to the reconstruction of the Heuneburg during the mudbrick wall period (adapted from [5]).

To obtain an overview of consumption practices, especially drinking practices, 133 ceramic vessels dating to the Late Hallstatt period from the Heuneburg were investigated. The primary goal was to carry out a qualitative comparison based on ORA. The samples were selected from collections (see S1 Tables for inventory numbers) at the Landesmuseum Württemberg (Stuttgart, Germany), the Landesamt für Denkmalpflege Baden-Württemberg in Esslingen and Tübingen, the Archäologisches Landesmuseum, Zentrales Fundarchiv in Rastatt and the Museum der Universität Tübingen (Germany). Vessels were selected from different occupation phases according to their techno-typological characteristics and/or stratigraphy (Ha D1 with some possibly belonging to the Ha D1/D2 phase, *n* = 72; Ha D2/D3, *n* = 7; Ha D3, *n* = 42 and unstratified Ha D, *n* = 12) and the three main areas of the site, namely the plateau (*n* = 67), lower town (*n* = 41) and outer settlement (*n* = 25) (Fig 2).

Vessels selected from closed contexts and, in ideal cases, from *in situ* contexts were our primary targets of analysis. Due to post-depositional processes, however, most of the finds had been relocated between their time of use and their discovery. To obtain a representative quantity of vessel shapes corresponding to the main questions of our project (i.e. vessels that are supposed to have been used during feasting practices) we also had to include these relocated finds in our study.

**Fig 3:**
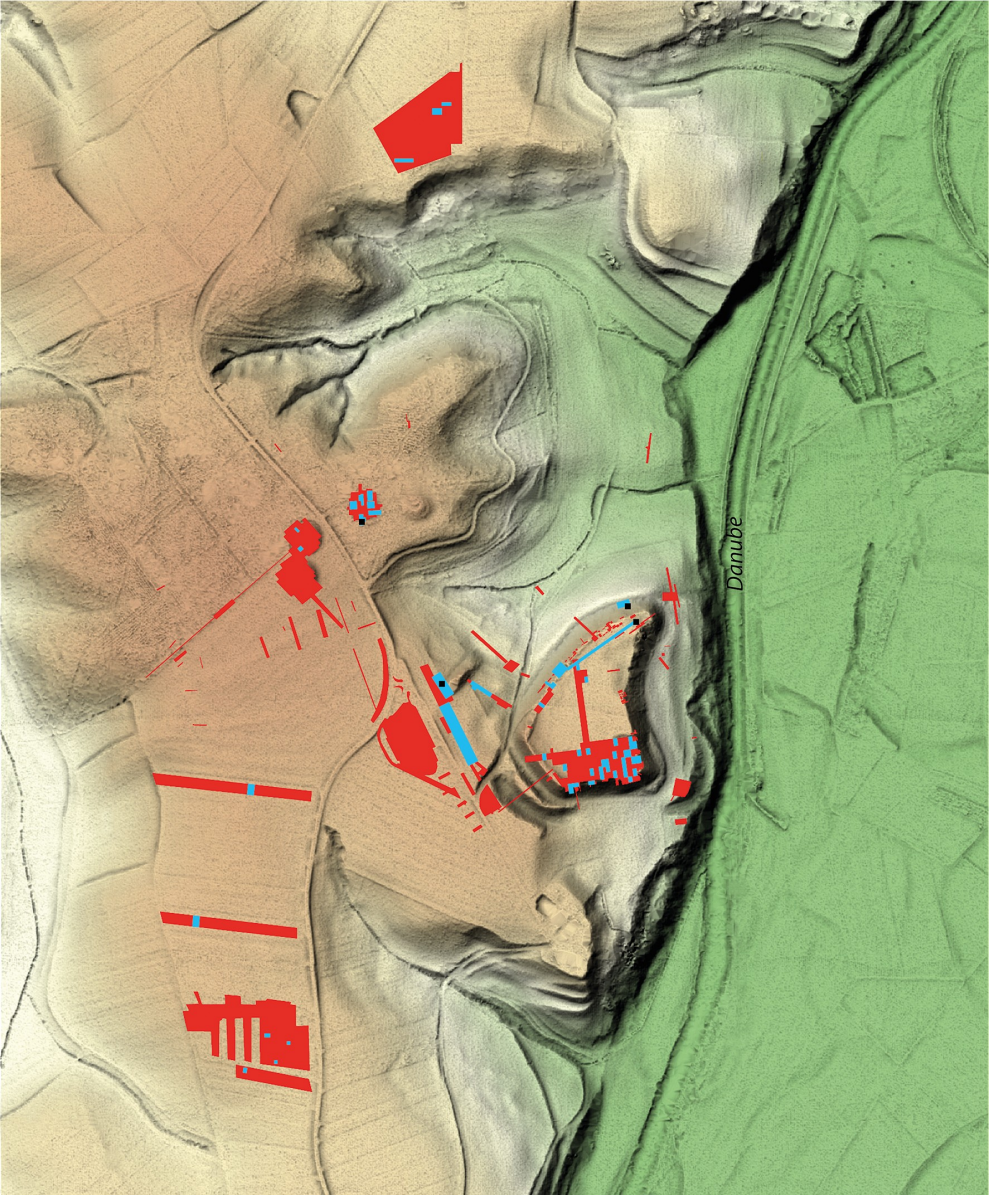
Surface mapping of the finds from old and new excavations at the Heuneburg plateau, lower town and outer settlements. Red: all excavated areas between 1950 and 2014. Blue: surface mapping of the contexts from which samples were selected for ORA. Map basis [22]. Black squares: control samples (see S1–S4 Figs).

The vessels from the plateau comprised 60 locally produced and 7 imported Attic vessels [7]. We selected them from two large pottery assemblages dating to Ha D1 (n = 19) and Ha D3 (n = 13). Additional vessels were chosen from various other contexts within the plateau (n = 35, Fig 3). We obtained 41 local pots and all 7 imported vessels from the archives of the old excavations carried out between 1951 and 1979. These vessels have been published in terms of their ware and vessel shapes but not their context of discovery, with the exception of certain outstanding pottery assemblages whose context had previously been published. Among these are the *in situ* finds from the towers of the surrounding defensive wall and the ceramic inventories of particular households [8, 9]. Since the vessels recovered during old excavations are currently stored by ware and not by context, it was not possible to reconstruct other find assemblages besides the ones that were already published. Out of the published assemblages, the only one that could be located was the ceramic inventory of a Ha D3 household [8]. It provided 13 vessels for our analyses (HB-PL-106, 302, 303, 601, 605, 606, 612, 616–618, 621, 626A, 626B). None of the Attic imports belonged to the same contexts as the locally produced ceramics. In addition to the vessels from the old excavations, we selected 19 vessels from excavations carried out in 2015of a Ha D1 pit house filling found to the north of the plateau (HB-PL-001-019). Fragments of golden wires recovered from the same context, which was extremely rich in terms of the quantity of the finds, suggest a connection with the golden jewelry of the Bettelbühl grave mound [10].

With regard to the lower town settlement, we chose 41 locally produced and yet unpublished vessels from recent excavations (2004–2008, cf. preliminary reports: [11–20], Fig 3). Eleven vessels were found in contexts associated with an early phase of Ha D1, i.e. from ancient humus layers (features 1590 and 1109, n = 2), burnt settlement layers (features 1232 and 842, n = 3), a post-hole building with a preserved living floor level (feature 1870, n = 4) and the remains of a vaulted oven (feature 1819, n = 2). Ten vessels were recovered from the former settlement layers of the rampart in contexts related to an advanced stage in Ha D1 when the Heuneburg plateau was surrounded by the mud-brick wall (feature 1159: n = 9 and feature 1693: n = 1, respectively). In addition, we studied 15 vessels discovered in contexts from the lower town settlement in Ha D3. These features comprise a living floor level (feature 192, n = 3), pit house fillings (feature 84: n = 2; feature 455: n = 5; feature 178: n = 1), a burnt settlement layer (feature 1674, n = 3), and the filling of a trench (feature 2007, n = 1). Five vessels from various contexts were selected because of their shapes in order to increase the sample of Ha D3 vessels.

The outer settlement sample comprised only Ha D1 ceramic vessels. Fourteen samples were selected from vessels excavated between 1954 and 1981 [21]. Regarding the finds from these old excavations, our selection of vessels had to be based once again on shape rather than context. However, two of the four goblets (HB-AS-003 and 029) selected were found in the same feature, the filling of a shallow pit. A cist and a bowl (HB-AS-012 and 013) from the same ditch were also selected. Two coarse vessels (HB-AS-034 and 035) were likewise found together in a ditch and were included in the analysis. In addition, archaeological investigations conducted between 1995 and 2005 provided 11 coarse ware vessels that might have been used as storage jars or even cooking pots. These vessels were recovered from various contexts at the outer settlement and were selected for analysis in order to provide examples from different locations within the extensive settlement area (Fig 3). These vessels were found in several features including the fillings of pit houses, vessel deposits, a storage pit, the foundation trench of a fence, and ditches.

The imported Attic wares (*n* = 7) were thought to have played an important role in the consumption and/or preparation of beverages. As shape and/or function equivalents, local fine (*n* = 89) and medium fine (*n* = 10) wares were investigated. Among the 80 local fine low forms considered to have been used as drinking vessels, 4 wheel-made bowls, 50 handmade bowls (comprising both small and middle/large forms), 7 beakers, 12 possibly Mediterranean inspired goblets, 2 miniature vessels and 5 undetermined small forms were sampled. Bowls could have been used for different purposes such as storage, preparation and as serving vessels. The serving vessels comprised 9 local fine high forms including 2 jugs, 6 bottles, and 1 cist. Bottles might have had a storage/preparation function related to liquid content(s), and a similar function is hypothesized for the 10 middle fine high forms tested, including 6 cone-necked vessels and 3 large vessels (Fig 1). To have a complete overview of the ceramic vessels used in food/drink consumption practices, 2 sieves, 2 funnels and 24 coarse vessels were selected and tested. The latter are associated with storage and/or preparation functions, including cooking.

### Sample treatment before GC-FID/MS

ORA analyses were carried out at the University of Tübingen in the Departments of Recent Prehistory and Geoscience, Germany. 1–2 g of potsherd were drilled following cleaning of the vessel surfaces to remove any exogenous lipids (Layer 2). The characterization of the lipid compounds present was based on the analytical results obtained from Layer 2. The ceramic powder collected during surface cleaning (Layer 1) was retained for potential additional analysis. Where it was possible to obtain sediment, soil samples were taken from recent excavation contexts on the plateau and in the lower town settlements to control for exogenous contamination. Samples were also taken from the external surface of the vessels selected from the outer settlement and used as controls, as no sediment was preserved during the old excavations (see S1–S4 Figs). Powdered sherds were solvent-extracted (Dichloromethane-Methanol, 2:1, *v*:*v*) by ultrasonication to target lipid compounds following established protocols [23, 24]. 50% of the total lipid extract were trimethylsilylated using *N*,*O*-bis(trimethylsilyl)trifluoroacetamide (BSTFA, 50 μL) and a catalytic reagent (pyridin, 4 μL) before analysis by GC and GC-MS. To target short-chain carboxylic compounds (insoluble in organic solvents), two other established protocols, KOH (1M) treatment with extraction in ethyl acetate [25] and BF_3_/BuOH/cyclohexane treatment with extraction in dichloromethane [26], were used. Some of these compounds are present in relatively high quantities in fruit products.

### GC and GC-MS analyses

The analysis of trimethylsilylated samples was performed by Gas Chromatography (GC) and GC-Mass Spectrometry (GC-MS) using an Agilent Technologies 7890B GC System series chromatograph including Agilent Technologies Capillary Flow-Technology Three-Way Splitter Kit coupled to an Agilent Technologies 5977A MSD and FID. The analyses were carried out using helium as a carrier gas, with a split/splitless injection system (Gerstel Multi-Purpose-Sampler and Gerstel Cold-Injection-System 4), operating in the splitless mode with a purge flow of 3.0 ml min^–1^ and a constant pressure at the head of the column of 8.6667 psi. Samples were analyzed using an Agilent J&W DB-5HT-column (15 m × 0.32 mm i.d.; 0.1 μm film thickness) and divided in two equal parts using 0.18 mm non-coated, deactivated silica capillary columns (0.66 m splitter-column to FID/ 1.52 m splitter-column to MSD) with the Three-Way Splitter Kit. The inlet temperature was ramped from 30°C to 240°C at 12°C s^-1^ (held isothermally for 5 min) and then increased to 350°C at 12°C s^-1^ (held isothermally for 10 min). The temperature of the oven was set at 50°C for 1 min followed by an increase to 100°C at 15°C min^–1^, then to 240°C at 4°C min^–1^ and to 380°C at 20°C min^–1^ (held isothermally for 7 min). Mass spectra were acquired using electron ionization at 70 eV and obtained by scanning between m/z 50–950 in 1.562 s. The interface and the ion source temperatures were 300°C and 280°C, respectively. The temperature of the FID detector was fixed at 340°C. Mass spectra were matched against authentic standards (saturated and unsaturated triglycerides, fatty acids, *n*-alkanes, short-chain carboxylic compounds), published literature [27–30] and the National Institute of Standards and Technology (NIST) library, 2014 edition.

## Results and discussion

### ORA results and substance interpretation

Molecular biomarkers which can be diagnostic of the original content (lipids and/or short-chain carboxylic acids) were preserved in most of the samples investigated (122 out of 133; 116 samples containing ≥5μg/g of lipid). Several molecular families were identified including fatty acids, *n*-alcohols, *n*-alkanes, ketones, long chain esters, triglycerides, terpenes and short-chain carboxylic compounds, pertaining to over 10 natural organic products having an animal or plant origin. Details of the analytical results are provided in the supplementary information (Tables A-F in S1 Tables).

#### Fatty acids, triglycerides and ketones of animal origin

**Fig 4:**
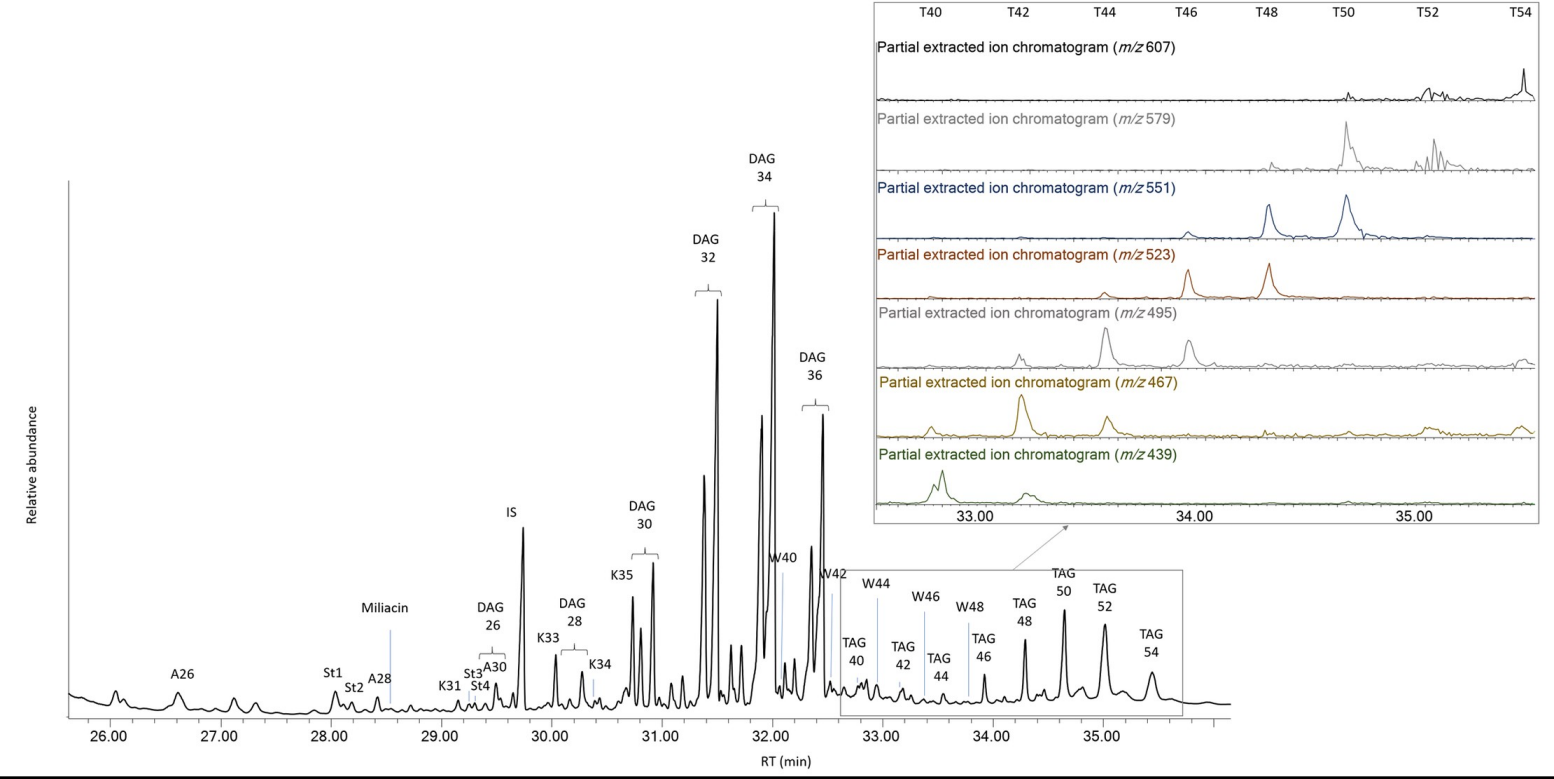
Chromatogram showing the molecular constituents of waxes, millet and dairy products in a local fine ware bowl from the plateau (HB-PL-017). Ax = n-alcohols, Kx = ketones and Wx = long chain esters with x carbon atoms. St1 = Cholesterol; St2 = Dihydrocholesterol; St3 = β-Sitosterol; St4 = Stigmastanol. DAG = Diglycerides; TAG = Triglycerides.

**Fig 5:**
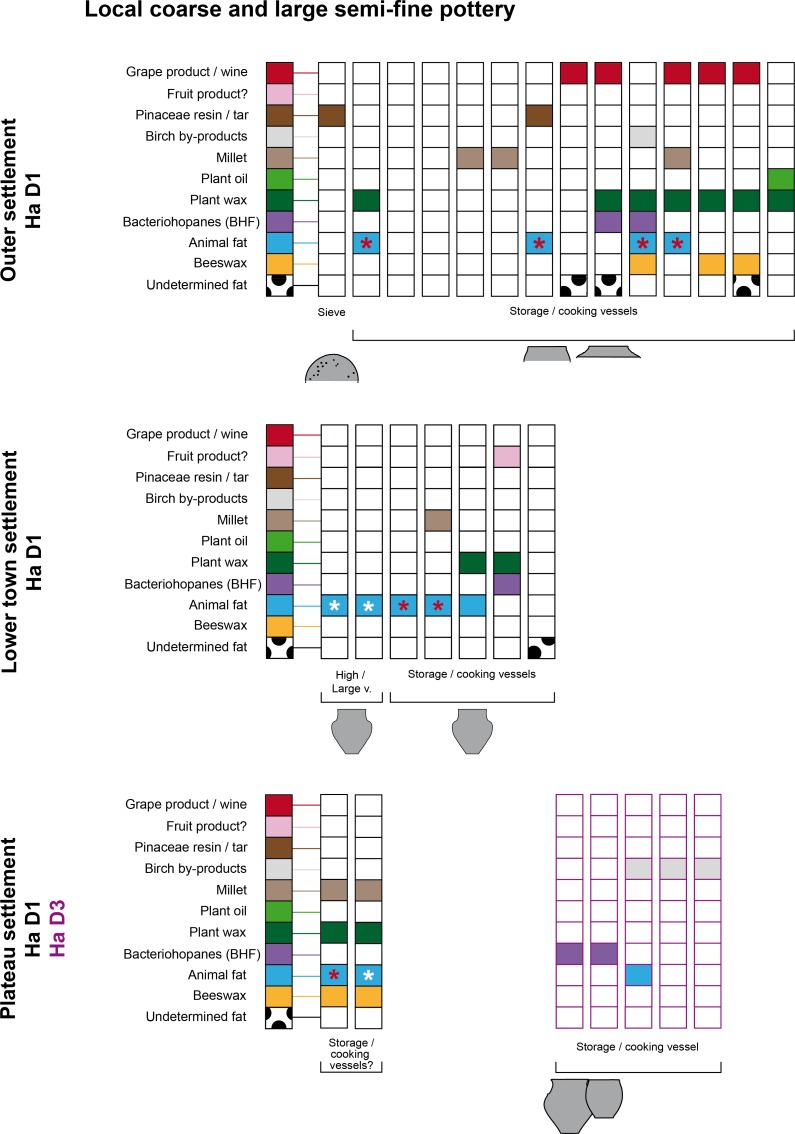
Organic substances identified in the semi-fine and coarse pottery from the Heuneburg according to the different settlement contexts of the Late Hallstatt period. * White = dairy product and *red = heating treatment of animal fat.

**Fig 6:**
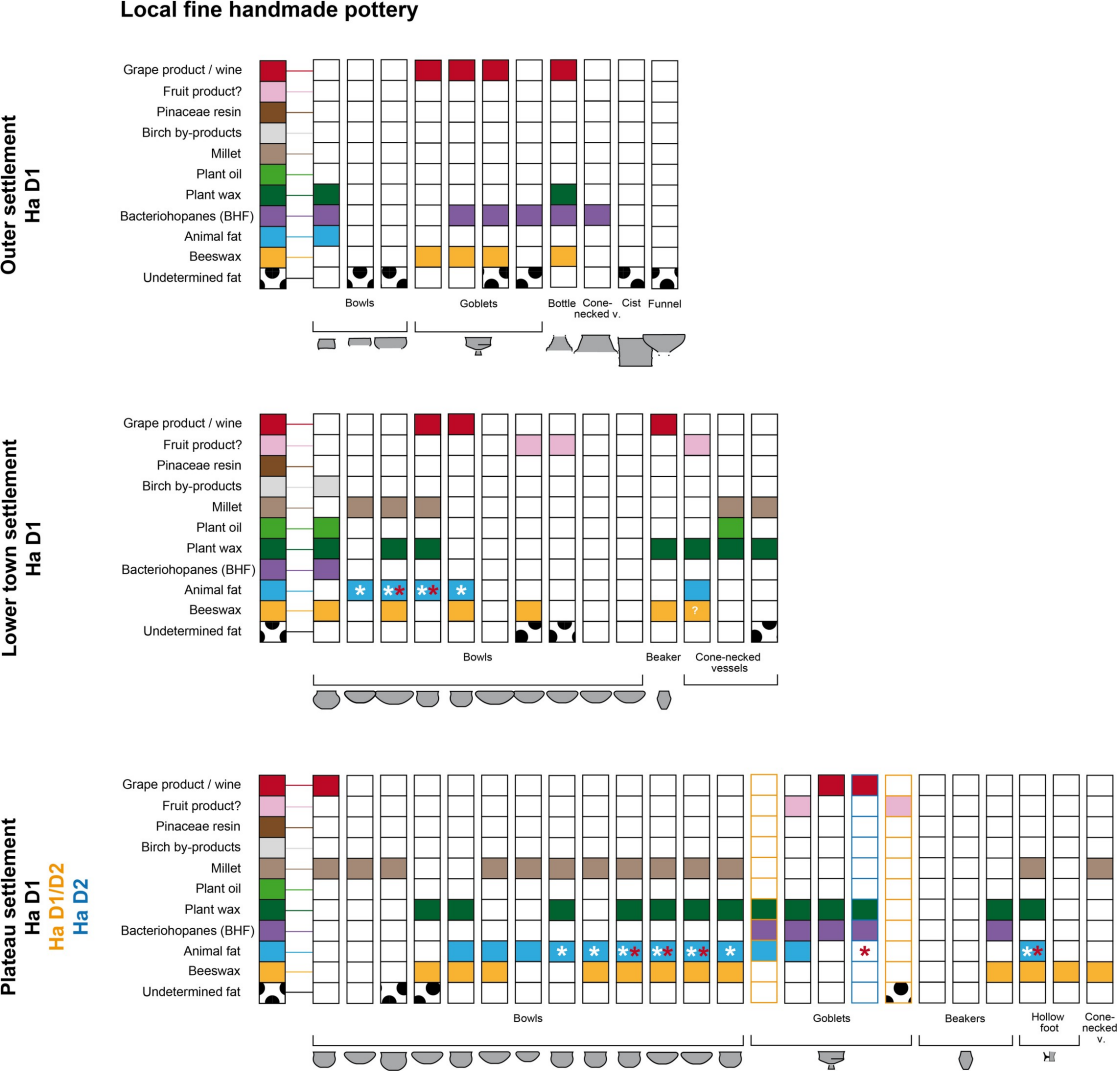
Organic substances identified in the fine pottery from the Heuneburg according to the shape and the contexts of Ha D1, Ha D1/D2. * White = dairy product and *red = heating treatment of animal fat.

Results show a significant use of animal fats in the selected vessels (n = 42; 32%). Their presence was characterized by the distribution of saturated fatty acids, mono-, di- and triglycerides (TAGs) that contain up to 54 carbon atoms [29, 31, 32] (Fig 4). Based on molecular markers, it was at times difficult to identify the animal origin for samples containing only high TAG signatures since hydrolysis from natural and anthropic degradation preferentially affect the shorter TAGs [33]. However, the identification of a large distribution of TAGs containing 40 to 54 carbon atoms points to the presence of dairy products [29, 34] in at least 18 vessels. The presence of dairy TAGs does not exclude the potential presence of animal adipose fats as part of mixtures or from vessel reutilization. A series of odd- and sometimes even-numbered ketones (K31 to K35) including asymmetric ketones (mostly K33 and K35) were identified in 14 vessels. Asymmetric ketones are markers of thermal transformation of animal fats and can be formed during roasting (>300°C) or repetitive use at more moderate temperatures [35, 36]. For this reason, they are generally used to determine if ceramics have been repeatedly used as cooking pots [36–39]. This hypothesis is plausible for six coarse ware vessels and one semi-fine ceramic vessel (Fig 5) often associated with this function. However asymmetric ketones were also found in fine low forms (n = 7), including bowls, a goblet and a hollow foot vessel (Fig 6). Their shape and thermic properties preclude their use for cooking as they are not suitable for strong heat treatment. A secondary heat treatment of the ceramic related to the site conflagration does not appear to be a direct cause of ketone formation, which is not always identified in vessels from similar excavated contexts. No evidence of sooting was observed on the surface of most of these vessels, but carbonized residues were identified in 3 semi-large bowls (HB-VB-010, Table B in S1 Tables; HB-PL-016 and -017, Table D in S1 Tables) and perhaps in one S-shaped bowl (HB-PL-006, Table D in S1 Tables), which could potentially show successive heating of these vessels at moderate temperatures. However, it is important to note that the presence of the surface carbonized residue and asymmetric ketones were generally not correlated in the Heuneburg vessels. Recent work suggests that asymmetric ketones can result from a heat-coating step using an animal fat. The heightened surface temperature of the vessel is capable of forming long-chain ketones. In this case, a heat-coating step with milk would usually be rejected due to milk’s known rapid rate of degradation, although it is possible that other animal fats were used [40]. The application of this practice is difficult to prove for the Heuneburg assemblage tested because we could not link the presence of asymmetric ketones to a specific techno-typological category, even though they are present in higher proportions in the Ha D1 bowls (5/26). What is interesting here is the systematic presence of dairy products when asymmetric ketones were identified in Ha D1 bowls (n = 5) (Fig 6). This combination could suggest the use of such vessels for (i) the consumption of both cooked/roasted meat and dairy products or (ii) the processing of dairy products [37]. Indeed, the preparation of dairy products such as yoghurt or cheese may require heat treatment [41] and could plausibly account for our findings (repetitive use at moderate temperatures).

#### Fatty acids of plant origin

Significant quantities of oleic acid (oleic acid ≥ stearic acid) could suggest the presence of oily plants or plant by-products in 6 vessels (Tables A-B, F in S1 Tables). High quantities of oleic acid, together with other C18 unsaturated markers and palmitic acid as the major fatty acid are considered markers of plant oils. High oleic to stearic acid ratios are extremely rare in archaeological samples. Unsaturated fatty acids are more susceptible to degradation processes than saturated fatty acids, primarily through oxidation [42]. When present, their identification has been associated with plant oils [43]. The ratio of palmitic to stearic acid in the 6 samples is higher than 3:1 and could also be indicative of a plant oil (higher than 2:1) [43], even if this argument is less persuasive for archaeological residues due to the differential degradation processes of fatty acids [42]. Based on the local oily plants in southwestern Germany documented by the archaeobotanical data [44], possible sources could be the exploitation of linseed, opium poppy and camelina. Another potential source could be the oily part of cereal by-products which were also present at the Heuneburg (barley, spelt, millet, emmer, oat and einkorn), though the much lower quantity of oil found in cereals does not support this hypothesis.

#### Esters, n-alkanes, n-alcohols and fatty acids of beeswax

**Fig 7:**
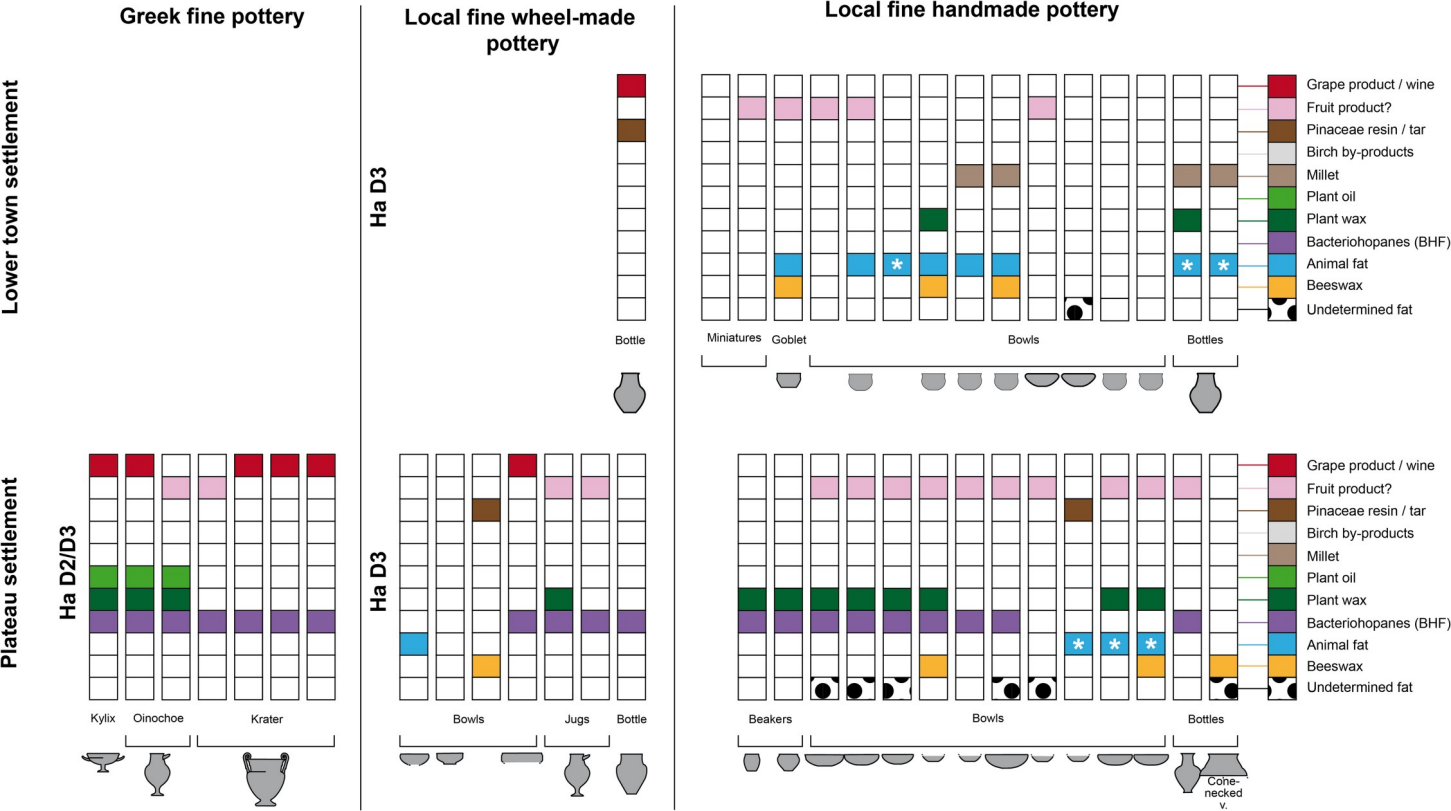
Organic substances identified in the fine pottery from the Heuneburg according to the shape and the contexts of Ha D2/D3. * White = dairy product.

Beeswax was identified in a significantly large number of local vessels (n = 37; 29%) (Figs 4–7, Tables A-E in S1 Tables, S5 Fig), demonstrating extensive exploitation. The presence of this beehive product was characterized by long chain palmitic esters with an even number of carbon atoms ranging from C40 to C50, in combination with their hydrolysis products (palmitic acid and even-numbered *n*-alcohols comprising 22 to 34 carbon atoms), a series of odd-number *n*-alkanes (C_27_ major) and the frequent presence of saturated long chain even-numbered fatty acids (C_22:0_ to C_28:0_) [45–47]). Beeswax is a versatile material with applications for diet, body care, art and technology [47–49]. The presence of beeswax could also suggest consumption of incompletely filtered honey, which comprises mainly fast-decaying saccharides.

#### Esters of plant wax

Waxy products in combination with *n*-alkanes, *n*-alcohols and especially esters differing from those found in beeswax, generally denote a plant origin [32, 50]. The presence of long chain palmitic esters (C30-C42) and/or stearic esters in 48 samples (36%; Tables A-F in S1 Tables, S5 Fig) suggests different plant waxes which can be found in the leaves of various fruits, vegetables and cereals [50]. However, it is difficult to precisely identify the plant species because these molecular families are present in epicuticular waxes with little differentiation between species, at times distinguishable only in their relative proportions. The possibility of mixtures of plant by-products further increases complexity [51]. Their presence could result from the consumption of plant-by products identified in the botanical records such as oily plants, cereals, legumes (lentil, pulses, pea) and fruit [44].

#### Common millet

The presence of miliacin in 20 vessels, a diagnostic triterpenoid marker, shows a significant use of common millet (15%) [28, 52] which can be consumed as whole grains, porridge, cakes, or fermented beverages. The absence of wheat, barley and rye alkylresorcinols [53] from the pottery does not exclude these cereals from having been present in the vessels, as their absence could result from their lower stability or concentration compared to miliacin, leading to an over-representation of millet. Furthermore, the archaeobotanical record points to barley as the major cereal at the Heuneburg and in other parts of the southwest Germany [44].

#### Pinaceae resin and tar

Pine resin diterpenoids were detected in one bowl from the plateau and in one sieve from the outer settlement. A *Pinus* origin is suggested by the presence of seco-dehydroabietic acid, β and α isomers, isopimaric acid isomers, pimaric acid, sandaracopimaric acid, dehydroabietic acid and abietic acid. [30, 54–56]. Retene and methyldehydroabietic acid were also identified in two wheel-turned wares (bottle and bowls). Further hydrocarbon markers, namely 1-(10-Methylanthracen-9-yl)ethanone, 9-Methyl-10-phenylanthracene combined with dehydroabietic acid, were identified in a coarse vessel recovered from the outer settlement. Hydrocarbon and methyl diterpenes are generally formed by direct or indirect degradation of abietic acid following heat treatment and suggest the production of a Pinaceae tar [57]. Pinaceae tar was widely exploited in the Mediterranean area, whether for vessel coating or ship caulking [58, 59]. Pine resin can potentially be used for its hydrophobic proprieties but also for flavoring [48].

#### Birch bark tar

The presence of betulin and lupeol, the major triterpenoid biomarkers of birch bark [60], [61], other additional biomarkers (erythrodiol, betulinic acid, betulin ethers and betulin-28-caffeate) in combination with degradation markers including allobetulin, allobetul-2-ene and α-betulin I were identified in the surface residue of 3 coarse vessels from the plateau. These are characteristic of thermal degradation during tar production using a mild heat treatment *per descensum* [62]. The identification of birch bark tar confirms previous analyses conducted using TLC methods [63]. The homogenous layer of tar applied to the bottom of one vessel (HB-PL-110) suggests a coating (S6 Fig), but the function of the residues present in the internal and external surfaces of the other two vessels is still unclear. Two other samples, a coarse vessel from the outer settlement and, more surprisingly, a fine bowl from the lower town, also show the presence of triterpenic markers characteristic of birch bark tar (lupa-2,20(29)-diene, lupa-2,20(29)-dien-28-ol, lupenone, lupeol, 28-oxoallobetul-2-ene, 3-oxoallobetulane). However, the absence of several biomarkers, especially betulin, indicates a higher degradation. This may be explained by repetitive use/heating. The lack of visible residues of birch bark tar from the outer and the lower town settlements does not provide clear evidence for a similar functional interpretation. The tar could have been used to seal the vessels, or perhaps tar could have been stored or produced in these vessels, particularly in the coarse wares.

#### Hopanes: markers for a bacteriofermented beverage?

**Fig 8:**
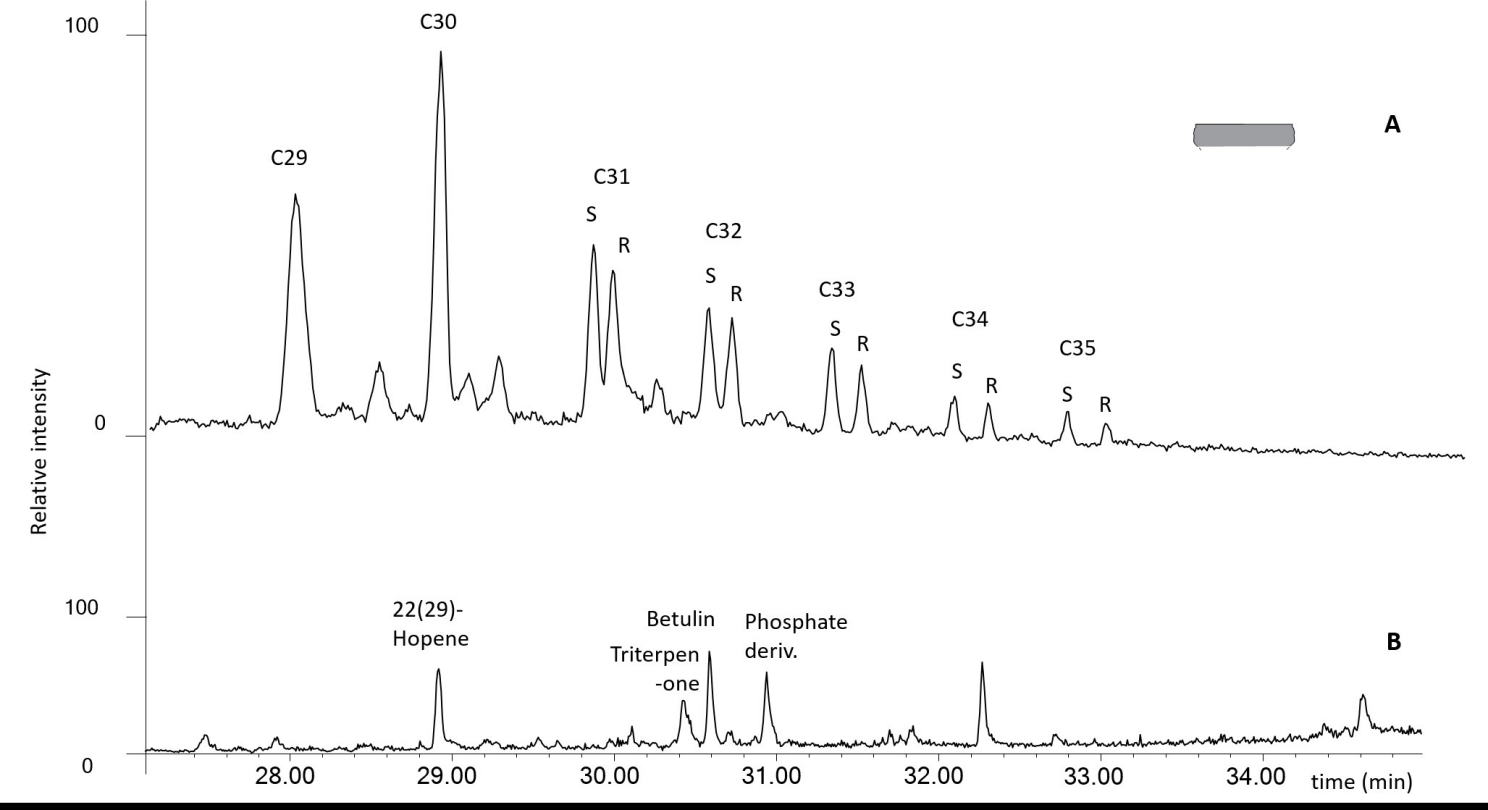
GC-MS selected ion monitoring (m/z 191) showing: A) hopane distributions (C29–C35) inside a wheel-made bowl (HBPL617) recovered from Heuneburg, plateau and B) soil control samples (HBSED06) from the Heuneburg, plateau and lower town settlement.

In 46 vessels, triterpenes from the hopane family were characterized by a base peak at *m/z* 191 and molecular ions (M+.): 398 (C_29_H_50_) and 412 (C_30_H_52_), and occasionally 426 (C_31_H_54_), 440 (C_32_H_56_), 454 (C_33_H_58_) and 468 (C_34_H_60_). Although their mobility in soils and their migration in potsherds is probably limited [64], hopanes are ubiquitous compounds in the environment. They can also result from bacterial activity and are regularly observed in wet soils, mostly in peat environments. At the Heuneburg, we excluded exogenous contamination of the sherds by analysing control samples, in particular soil samples taken from the feature contexts (Fig 8 and S1–S4 Figs). In archaeological contexts, hopanes can indicate the presence of various products such as bitumen [65, 66] but also fermented beverages [67]. In the latter, fermentation bacteria *Zymomonas mobilis*, are known to develop a membrane rich in hopanoids to resist ethanol stress and low pH levels [68]. At the Heuneburg, the hopane assemblage is more consistent with bacteriofermented product(s) than with bitumen or marine shale (absence of gammaceranes or methylated hopanes). It is theoretically possible that hopanes are present due to bacterial activity on the sherd immediately following deposition and can become incorporated in the organic residue. However, when present in the Heuneburg assemblage tested, bacteriohopanes are consistently associated with specific organic substances, namely plant by-products (especially with plant wax, in 50% of all cases) and beehive products, whereas there is hardly any evidence for their association with animal fats. Furthermore, hopanes were found mostly in drinking and serving vessels, in particular in the Attic wares and goblets, and were rarely found in coarse vessels. Without excluding the possibility of other fermented beverages which could include various plant or bee by-products, a possible interpretation is the production of beer from cereals, which is further supported by high quantities of botanical evidence from the Heuneburg contexts [44].

#### Short-chain carboxylic compounds: fruit products?

**Fig 9:**
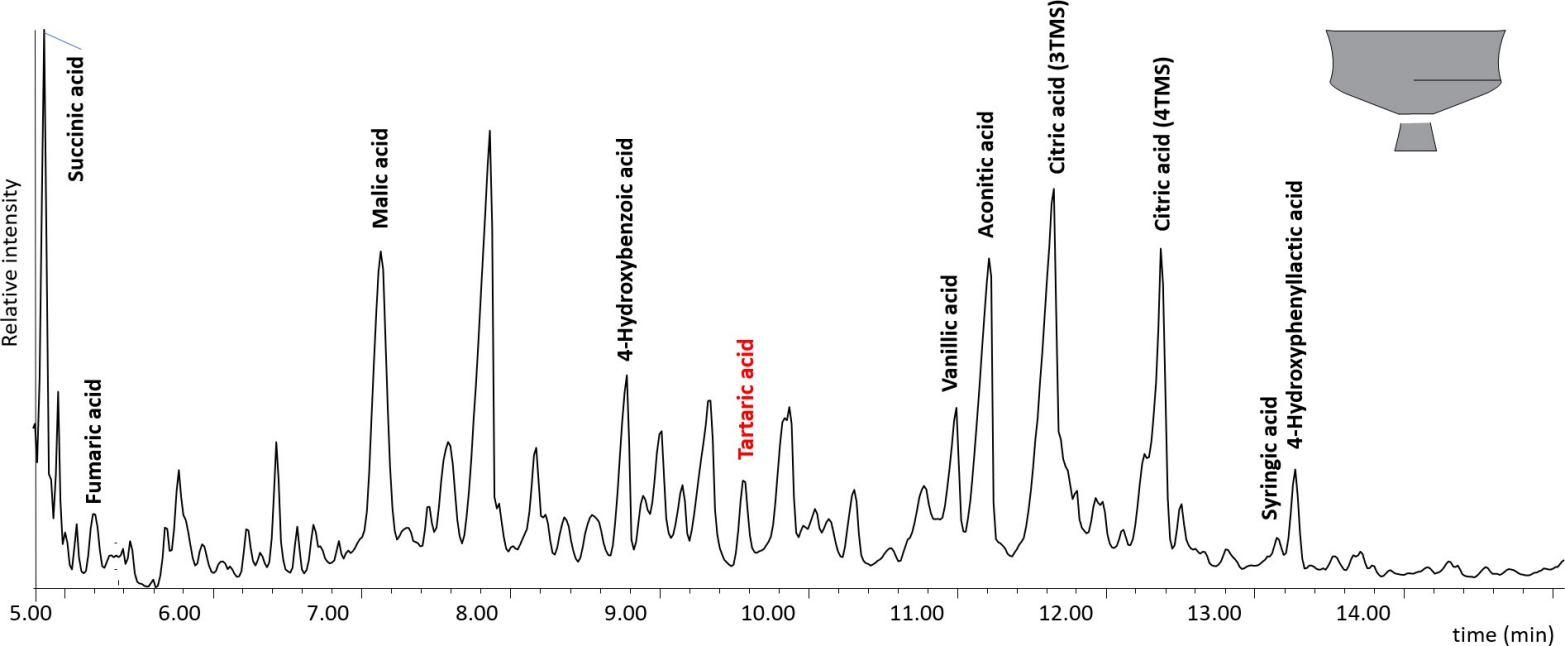
Chromatogram showing the molecular constituents of wine (following KOH-1M treatment and extraction in ethyl acetate [25]) in local fine goblet from the plateau (HB-AS-27).

The presence of succinic, fumaric, malic, and at times tartaric and syringic acids was identified in 53 vessels, but not in the soil control tested (S4 Fig). This assemblage of short-chain carboxylic acids and phenolic compounds suggests the presence of fruit products. Tartaric acid, identified in 24 vessels, is usually interpreted as a biomarker for grape products/wine because of its higher concentration in grapes [25, 26, 69–71] compared to other fruits available in Europe during the Early Iron Age. When present in the Heuneburg vessels, tartaric acid was mostly identified together with other short-chain carboxylic compounds including succinic acid (Fig 9, Tables A-F in S1 Tables), which are known wine fermentation markers [25, 26]. Malic, succinic and fumaric acids were also identified in the absence of tartaric acid in some vessels, suggesting the presence of fruit products other than grape. Grape wine consumed at the Heuneburg was probably imported from the Mediterranean as there is a near absence of evidence of grape seeds in Central Europe. Thus, the exploitation of the local wild vine is not supported. Unlike Mediterranean contexts, there is no evidence of winemaking (e.g. pressed grapes) in the Central European Early Iron Age [72–74].

### Consumption practices according to techno-typology of the vessels

Early Celtic consumption practices were the subject of one of the earliest organic residue analyses on prehistoric pottery in Europe, conducted by Rolf Rottländer in Tübingen in the 1980s and 1990s [75–77]. These first results were important for further methodological developments. Rottländer tested 103 sherds from the Heuneburg and found possibly tarry and fatty substances. Although the analytical protocols and techniques available at the time may have influenced the interpretation of fatty substances, the tests showed a very good potential for the preservation of organic residues and strongly suggested the presence of markers of conifer and birch bark tars [63, 75]. The analysis of archaeobotanical remains in bronze vessels has also already yielded important insights into consumption practices [78]. Pollen derived from visible residues inside bronze vessels was studied from five high-status burial contexts (Heuneburg-Hohmichele, Heuneburg-Speckhau Tumulus 6, Hochdorf, Glauberg, Niedererlbach). In each context, the evidence from the pollen assemblages was interpreted as indicating the presence of mead inside the vessels (cauldrons, pitchers, a ladle), which could have been produced by soaking a large piece of honeycomb in water [78–83]. Only one of the two jugs from the Glauberg burial seems to have contained a clarified and decanted mead. These results pose an important challenge to the notion that Early Celtic elites preferred consuming wine as a means of demonstrating their high status. However, a combination of recent archaeobotanical and organic residue analyses on visible residues in the bronze cauldron from the princely tomb of Lavau indicates that the vessel contained grape wine which was sweetened by putting a honeycomb or a block of beeswax inside, as the latter was preserved as a yellowish block at the base of the vessel [84]. This new find raises the question of potential mixture(s) which have to be considered in future studies.

In the following section, the residues identified in the pottery from the Heuneburg settlements will first be discussed in terms of the techno-typological categories of the vessels.

The Attic sherds from the older excavation of the Heuneburg [7] had unfortunately been stored in plastic bags for several decades before the application of ORA. Quantitative and qualitative studies of the lipid fraction were limited due to coelution and high levels of phthalate contamination. Despite these difficulties, fermented substances were identified in the 7 imported Attic pottery vessels, which included 4 kraters, 2 oinochoe and a kylix. Hopanes, probably resulting from bacteriohopanoid-fermentation (BHF) of a plant or bee product were identified in all of these vessels. Grape wine was found in 5 of them (3 kraters, 1 oinochoe and 1 kylix). These results are consistent with a function related to feasting for these Mediterranean wares (Fig 7). It is also important to note the presence of plant oil and wax in the oinochoe and the kylix, which could be related to a fermented beverage(s) (cereal and/or mixtures with other plant types, possibly herbs) or the consumption of another, not necessarily alcoholic, plant-related drink (reuse).

Among the postulated drinking vessels from the category of local fine wares (goblets, beakers, bowls, and other small vessels), the consumption of fermented beverages was also very important (31/80, 39%), particularly for goblets (10/12, 83%) and beakers (4/7, 57%). Fermentation products, including imported wine (n = 5) and/or BHF products (n = 9), were present in most of the postulated Mediterranean inspired goblets (10/12), which are generally associated with the Ha D1 and HaD1/D2 contexts at the Heuneburg [21] (Figs 1 and 5). However, they were not always present as unique substances, as indicated by the presence of beeswax (3/4), plant wax (5/7) and/or animal fat (3/7, including one cooked). Wine (n = 1), BHF products (n = 3) and plant waxes (n = 4) were also identified in most of the 7 beakers when a residue was retained (4/5). Beeswax was only identified in two beakers dating to Ha D1. When considering the bowls tested, the percentage of fermented beverages including wine (n = 6) and BHF products (n = 11, often associated with plant wax) is less important (15/50, 30%) and supports the postulated polyvalence of this vessel category. Indeed, animal fats are the main product identified (23/50 bowls, 46%), followed by beeswax (17/50, 34%) and millet (16/50, 32%). In particular, dairy products were consumed from at least 14 bowls (28%). The dairy products and millet identifications were never found together with BHF, showing an alternate use–possibly for some kind of millet porridge. The context of discovery and possibly the vessel shape could also explain the difference in content. Indeed, the larger bowls are not always adapted for drinking practices and, at the Heuneburg during Ha D1, they are never associated with wine or BHF.

Among the high local fine wares (jugs, bottles and the cist) thought to contain a liquid or semi-liquid content and whose function has been associated with serving, fermented beverages are the main product identified (6/9). BHF products were present in the jugs and the bottles from the plateau, whereas wine was identified in the wheel-turned bottle recovered from the lower town. Both fermented products were identified in the handmade bottle from the outer settlement, together with beeswax and plant wax. However, the two handmade bottles from the lower town contained millet and dairy fat. Regarding the special forms, no clear results were observed for the cist and the funnel, but pine resin was identified in the sieve and may have been filtered. Pine resin could also have been applied on the sieve for its hydrophobic properties.

Among the vessels with assumed functions for storage, food and/or drink preparation (local coarse vessels, cone/high-necked vessels, and large semi-fine pots), a wide diversity of products was identified including oily and waxy plant by-products, millet, plant tars, fermented beverages, beeswax and animal fats (with some evidence for cooking), which include dairy products. The variability in content is related to the context of discovery, which will be discussed below. However, it is important to note that beeswax and millet were found in half of the cone-necked vessels analyzed (3/6) and birch bark tar was mostly identified in local coarse vessels. The presence of pine tar and birch bark tar in two coarse vessels could possibly be linked to a coating function for the large storage vessels. The rare association of plant oil, millet and animal fats together with fermented products (wine and/or BHF) seems to distinguish at least two specialized uses for coarse vessels: one associated with food preparation and the other with fermented drinks.

### Transformation of practices and spaces of consumption during the Early Iron Age at the Heuneburg

Early Celtic feasting practices, their relation to different parts of Celtic society and particular spaces within settlements, as well as their respective changes over time have been intensively discussed in recent decades. Debates surrounding these topics began in the late 19^th^ century with the excavation of elaborate drinking sets made of ceramics and/or bronze in Early Iron Age tumulus burials–also in the vicinity of the Heuneburg [85–87]. Sets of drinking vessels have since been found in many Early Iron Age burials including the famous princely graves (e.g. at Hochdorf [88–90], Kleinaspergle [91] and Vix-Mont Lassois [92, 93]) raising questions concerning the nature, functions and meanings of commensality within Early Celtic society. Feasting with imported Mediterranean dishes was generally considered as a distinctively elite practice in Early Iron Age societies (most prominently: [2]) and it is generally accepted that alcoholic beverages played a crucial role in Celtic society [86, 94, 95]. However, the character and stability of these elites has been under continuous discussion, as has the importance of feasting in the formation of their status and identity (e.g. [86, 89, 95–99]). Based on changes in the feasting equipment found in burials over time, transformations in feasting practices were identified. It has been increasingly emphasized in the last two decades that one should not assume a mere adoption of Mediterranean drinking practices, and instead consider the complexity of processes of appropriation [86, 90, 94, 95, 100–107]. A recent study of drinking practices in the Early Iron Age settlement of Vix-Mont Lassois seems to show that the conspicuous consumption of imported grape wine from Attic feasting dishes was limited to the Ha D2/D3 elites living on the plateau [71].

In contrast to the study on feasting practices at Vix-Mont Lassois, the stratigraphic resolution and the complex spatial differentiation of the Heuneburg between the plateau, the lower town and the outer settlement (at least during Ha D1), enable significant further insights. At the Heuneburg, we are now able to shed new light on temporal and spatial dimensions and their relation to dynamics of commensal practices. We are aware that the limited number of samples facilitates more qualitative rather than quantitative insights. Moreover, the abandonment of the outer settlement at the transition Ha D1/D2 and the appearance of imported Mediterranean pottery as well as locally produced wheel-made vessels in Ha D2/D3 limits the possibility of tracing developments and differences over space and time. Nevertheless, the rich corpus of Ha D1 and Ha D2/D3 samples from both the plateau and the lower town inform on transformations of consumption practices at two different spaces.

#### Practices of consumption linked to Ha D1 and D1/D2 vessels (6^th^ century BC)

During Ha D1 (i.e. before the final destruction of the mudbrick wall) and also Ha D1/D2 phases (Figs 4 and 5), the use of beeswax appears to have been prevalent (identified in 19 vessels, 38%), particularly in locally produced postulated drinking vessels (46%). This could suggest a high consumption of beehive products as a flavoring/sweetening agent or the use of beeswax as a sealant (waterproofing). The latter could be envisaged for the bowls excavated from the plateau, which often contained beeswax. The absence of beeswax in imported wares suggests a specific practice related to local vessels. During Ha D1, fermented beverages including wine were identified and consumed in local handmade ceramics across all of the different status-related contexts at the Heuneburg (plateau, lower town, and outer settlement). This suggests that a trade/exchange of Mediterranean products was already established before the presence of almost all Mediterranean ceramic imports at the Heuneburg. Wine was also detected in a vessel (HB-VB-064, Table B in S1 Tables) from a layer older than the construction of the monumental gate, which is thought to be contemporaneous with the construction of the mud brick wall. Furthermore, wine was not only identified in fine wares but also in coarse vessels from the outer settlement, suggesting storage and/or additional practices such as the preparation of wine-based drinks or food. With regard to drinking vessels of the Ha D1 phases, fermented beverages (wine and/or BHF) are sometimes identified together with other substances, especially animal fats. This unusual finding could indicate vessel re-use, multifunctionality or special recipes even if the consumption of fermented animal/dairy products cannot be totally excluded. The function of goblets and beakers is clearly associated with fermented beverages. Beeswax was also identified in the goblets found in the outer settlement, whereas animal fat, plant wax and oil were only found in goblets from the plateau. This could suggest a greater versatility in the use of goblets on the plateau.

Detailed examination of the pottery from the outer settlement revealed the highest proportion of wine (39%) and BHF products during Ha D1 (35%), which indicates that this extensive space was important for the consumption of local and imported fermented beverages (Fig 7). Such substances were not only identified in fine wares (mostly goblets) but were also present in coarse vessels, a cone-necked vessel and a bottle, indicating the storage and/or the preparation of fermented products (Figs 4 and 5). Moreover, there is an interesting correlation between beeswax and grape wine in the outer settlement, in particular in the fine wares, which might possibly indicate a systematic flavoring/sweetening of wine with a bee-product. Millet, plant wax, plant oil and cooked animal fats are also sometimes associated with coarse vessels recovered from the outer settlement, consistent with multiple uses of such vessels. Coarse wares could have been used for the storage/processing of fermented products and/or food preparation.

**Fig 10:**
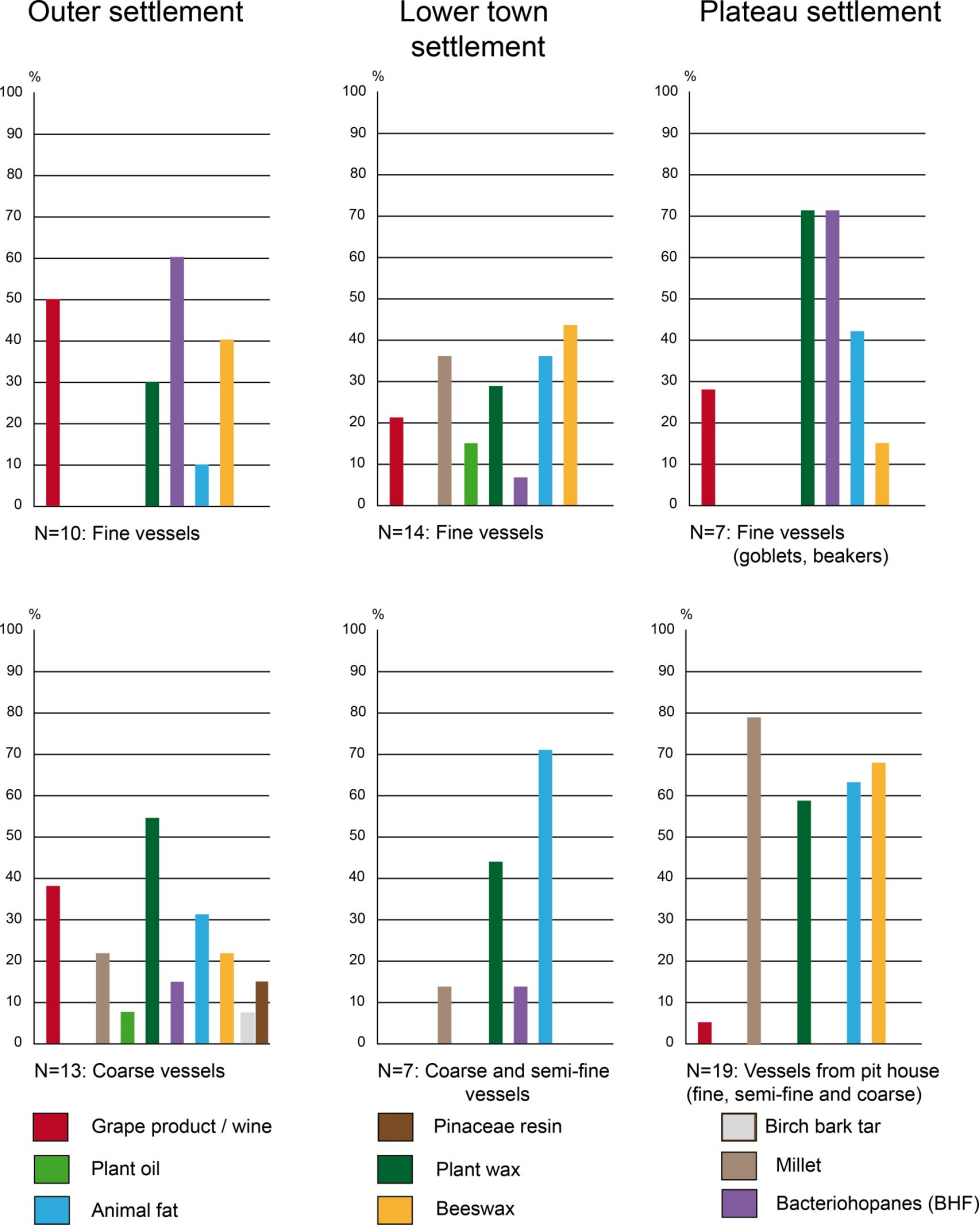
Organic substances identified in the pottery according to the spaces of consumption during Ha D1 and D1/D2 at the Heuneburg.

The use of bowls as well as fine and semi-fine high forms from the plateau and the lower town settlements for the consumption of fermented beverages is far less obvious, as indicated by the rare presence of BHF. On the plateau settlement during Ha D1, the bowls (n = 13), the hollow foot vessels (n = 2), and the large vessels (n = 3) were never associated with fermented products, except wine in one sample. The shared context of discovery of these finds seems to explain this particularity better than a shape-related function (sizes and shapes). Indeed, these ceramic finds, together with associated animal bones and metal finds (including gold fragments), were an important part of the fill material from the same pit house [10] (usually linked to activities in a nearby area). Activities related to food consumption and/or preparation could be specific to this intra-plateau area (Fig 10). Indeed, millet, dairy and beehive by-products were the most dominant residues in the vessels from this context. The combination of thermal treatment markers with a dairy molecular profile also suggests cooking and/or dairy processing.

Results obtained for the pottery from the lower town settlement show the only evidence of plant oil in fine wares during Ha D1 (one bowl and one cone-necked vessel, Fig 6). Unlike the pottery from the outer settlements, the coarse and semi-fine vessels from the lower town settlement are mostly related to the preparation/storage/consumption of food, including millet, animal and waxy plant products. The processing of dairy products could also potentially be present during Ha D1. It is interesting to note the presence of fermented beverages including wine in three bowls and one beaker. It is also the single context where wine and milk products were identified together in bowls, suggesting reutilization of these vessels.

#### Practices of consumption linked to Ha D2/D3 and HaD3 vessels (late 6^th^ to early 5^th^ century BC)

**Fig 11:**
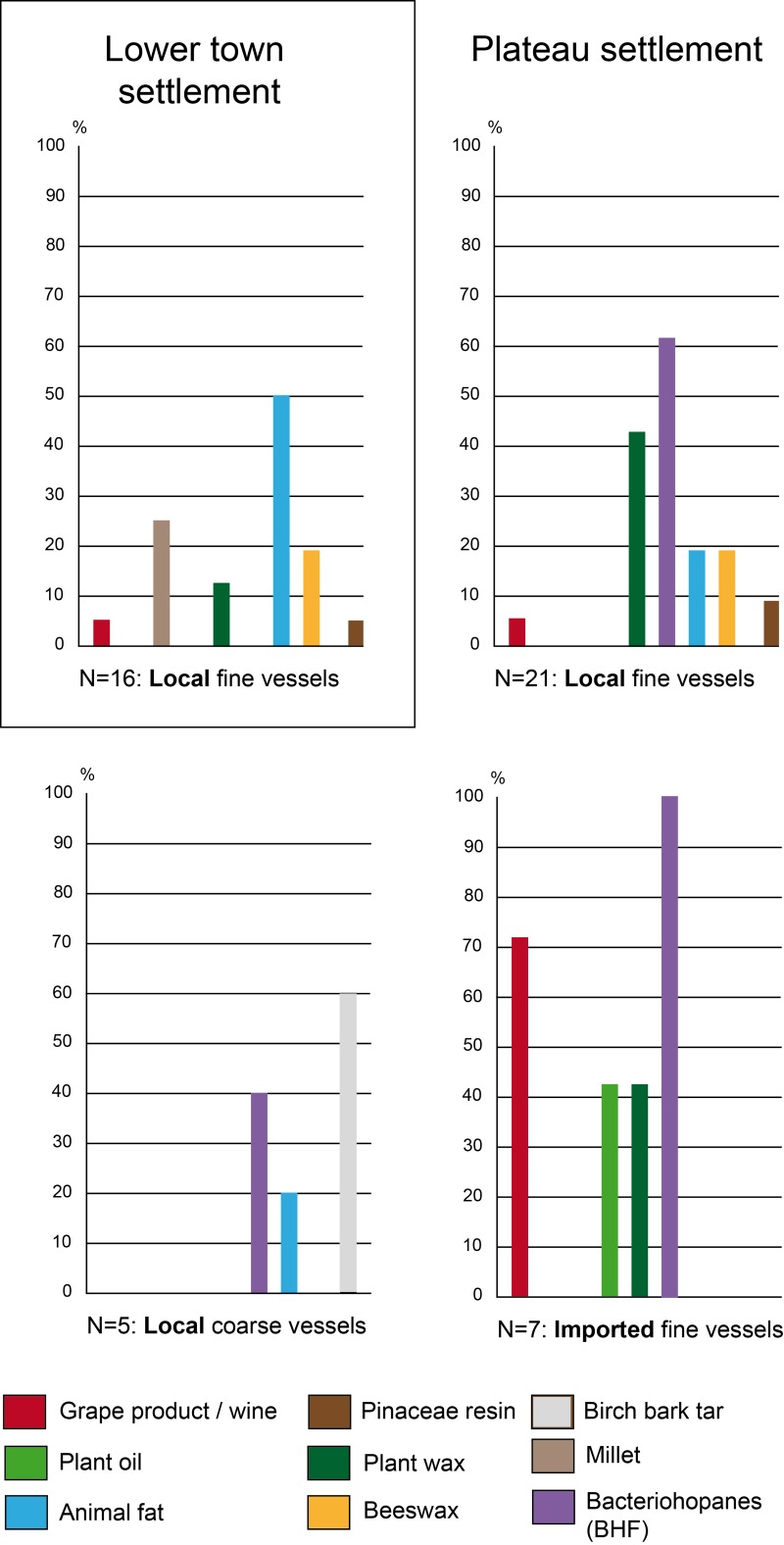
Organic substances identified in the pottery according to the spaces of consumption during Ha D3 at the Heuneburg.

During Ha D2/D3 or Ha D3 (all local wares), a specialized use of fine wares and possibly also of coarse vessels seems to have appeared on the plateau and in the lower town settlements (the outer settlement is not occupied at this time). Indeed, animal fats were never identified together with fermented beverages (wine or BHF products). This suggests a specialized use of the postulated drinking vessels which was not observed in the Ha D1 layers. Across the selection of vessels studied, the use of beehive products seems to decrease (17% of local vessels) and the fermentation products (wine and especially the BHF products) appear to be concentrated on the plateau settlement (Fig 11). In particular, BHF products were only identified in vessels from the plateau contexts (67%) and no longer in vessels from the lower town.

From Ha D2 onwards, wine is only identified in Greek imports and rarely in local wheel-made fine wares, which are considered to be local prestigious vessels. On the other hand, local handmade ceramics show an association with fruit markers, which could indicate the consumption of non-grape fruit beverage(s) due to the absence of tartaric acid. Moreover, cooking markers are absent in handmade wares from Ha D3 layers. Vessels recovered from the lower town settlement continue to be used for the consumption of animal and millet by-products during Ha D3. So far, no evidence for the consumption of millet was found on the plateau during this time.

Future research could investigate similarities and differences between our findings and the residues discovered in vessels from Hallstatt mortuary contexts, which would enable us to compare commensal practices of the living with those supplied for the deceased.

## Conclusion

By integrating archaeological and organic residue analyses we are able to shed new light on Early Celtic consumption practices and provide important initial insights into their complex transformation over time, which was certainly influenced in part by the dynamics of intercultural encounter with the Mediterranean. Our results gain even more importance within the framework of the discussions concerning Early Celtic commensality mentioned above. First and most importantly, the amount of imported pottery must not be taken as an indicator of the amount of imported Mediterranean wine. Secondly, the importation of significant amounts of wine started before the first clear evidence for the importation of Mediterranean feasting vessels (contra [95, 102]). The significant number of vessels containing wine residues during (and one even before) the existence of the mud-brick wall (Ha D1) shows that: i) we need to rethink the intensity of exchange in perishable products like wine from the Mediterranean to Central Europe during Ha D1 (cf. already [86]), ii) the consumption of wine was not limited to a certain space of the settlement during Ha D1 and it is therefore difficult to discern whether its consumption was reserved for the elite (as previously assumed) or accessible to all members of this community, and iii) wine consumption was initially not aimed at imitating Mediterranean feasting practices. Indeed, it was consumed from a large variety of vessels, which were also used for the consumption of other fermented beverages and/or food. It seems that the community which created and expressed itself and its power with the construction of the mud-brick wall [108, 109] was also formed and united by drinking imported wine–in both cases representing a “statement referencing a distant cultural source of power” [109]. Maybe labor was even mobilized by “work-party feasts” [94] that included wine consumption. The appearance of almost all of the imported Attic pottery in the late 6^th^ century BC (Ha D2/D3), after the end of the mudbrick phase, coincided with a clear change in function and meaning of wine consumption. Our results indicate that during Ha D3 wine was no longer consumed from local handmade pottery. Only imported Attic pottery and the newly produced local wheel-made pottery were now used for wine consumption. At the same time, other fruit by-product(s) were consumed from a large number of locally produced drinking vessels. Perhaps wine consumption became more conspicuous and possibly more restricted to imported and local wheel-made feasting dishes, as well as to a narrower part of the society (similar to a “diacritical feast” cf. [94]). This transformation could have been inspired by an increasing knowledge of Mediterranean drinking practices, hence the preferred use of imported kraters, jugs and drinking bowls, most of which have given evidence of wine. Certain actors within Early Celtic society seem to have managed to transform the meaning of wine by successfully limiting its consumption to certain vessels and spaces. These novel commensal practices seem to have served as a means of creating/enforcing their identities and to further establish/secure their position within society. It is most interesting to note that the findings from the Ha D2/D3-phases of the Heuneburg mirror the previous results for the contemporaneous Ha D2/D3 settlement of Vix-Mont Lassois [71]. This new relation between conspicuous wine consumption and social elites established in Ha D2/D3 extended into later Celtic society, when the Greek author Poseidonius reports that Celtic elites drank wine whereas the lower parts of Celtic society consumed beer (cf. [110]).

Another product associated with bacterio-fermentation (BHF), probably a bee- or a plant by-product, was found especially in the vessels from the plateau and the outer settlements. Our study demonstrates the consumption of such a beverage(s) during Ha D1, mostly in association with the Mediterranean style goblets that are often seen as an indication of the imitation of Mediterranean drinking practices [21]. However, their use for other fermented beverage drinking represents clear evidence for their creative appropriation [111]. The rare presence of BHF in coarse and cone-necked vessels could have been related to its preparation or storage, especially in the outer settlement. After the abandonment of the outer settlement (Ha D2/D3 and Ha D3), our results show that BHF beverage(s) are only found in vessels from the plateau, and always in association with imported pottery which had recently appeared. However, BHF beverage(s) are still identified in a large portion of the locally produced vessels studied, including handmade pottery.

Beehive products may also have played a role (as sweetener, sealant or possibly even as mead) in drinking practices, at least during the Ha D1 period, as suggested by the presence of beeswax with fermented beverages in goblets and beakers. These results confirm the importance of bee-products in the West Hallstatt area, which has already been noted for the settlement contexts at the site of Vix-Mont Lassois [71] and which was also indicated by the presence of honey/mead in the bronze vessels from Early Iron Age elite burials [81, 83, 84]. However, direct and unequivocal evidence for the presence of mead is as yet difficult to obtain using organic residue analysis. Hence, while there is a strong possibility that mead was present, in particular in light of the high quantities of bee products found in the vessels, we lack the data to assert it conclusively [71].

Animal fats and millet markers are rarely found in combination with fermentation markers (never during Ha D3) and are most probably linked to food practices. With regard to Ha D1 vessels, millet, animal fats (in particular dairy products), and beeswax are often identified together in the bowls and large vessels from a specific area of the plateau and the lower town settlements. Our results point to the importance of such products, possibly consumed together as some kind of millet porridge. A similar pattern is still observed in the Ha D3 vessels from the lower town settlement. However, the present study also shows that the consumption of beehive products seems to decrease, and millet is no longer identified in the vessels from the plateau. Other plant by-products may have also played a significant role in the diet as suggested by the presence of plant wax and plant oil, especially in the lower town settlement. The presence of dairy fats in a large number of differently shaped vessels points to the importance of these products in eating and drinking practices at the Heuneburg during the final Hallstatt periods. The importance of dairy products in the settlement contexts seems to be in accordance with some results of a recent proteomic study (a promising and fast developing new direction in obtaining dietary information from ceramics, see [112]) of six vessels from the burial context of Heuneburg-Speckhau Tumulus 17 [113, 114].

Specialized use could be attested for some vessels, such as Attic ceramics and local goblets/ beakers, which were used specifically for the consumption of fermented products (wine and/or BHF). Additional use(s), such as the consumption of other food products, were observed in local bowls and large vessels during Ha D1. The presence of food and fermentation products, sometimes in the same vessel, could indicate multi-functionality of vessels. Based on the vessels tested, an increase in the specialized use of drinking and serving vessels can be observed during Ha D3, when fermented beverages and animal fats were never identified together.

Finally, our study reveals some patterns related to the spaces of consumption. We show evidence for (i) the important and unexpected consumption and preparation/storage of fermented products (including wine) in the outer settlement, (ii) activities related to food consumption and/or preparation (dairy, millet and beehive by-products), which could be specific to the intra-plateau area during Ha D1, and (iii) a possible distinction of practices between the plateau (consumption of fermented drinks) and the lower town settlement (food consumption) during Ha D3.

## Supporting information

S1 Tables **A-F. List of samples including the analytical results and their interpretation arranged according to context.** Table A: Local vessels from the outer settlement during Ha D1. Table B: Local vessels from the lower town settlement during Ha D1. Table C: Local vessels from the lower town settlement during Ha D3. Table D: Local vessels from the plateau settlement during Ha D1 and D1/D2. Table E: Local vessels from the plateau settlement during Ha D3. Table F: Imported vessels from the plateau settlement during Ha D2/D3.
(XLSX)

S1 Fig Control sample from the plateau context (absence of hopanes). GC-MS selected ion monitoring showing: A: hopane distributions (m/z 191) inside wheel-made bowl (HBPL617) from the Heuneburg, Plateau context (Ha D3); B: absence of methylhopanes (m/z 205) inside wheel-made bowl (HBPL617) from the Heuneburg, Plateau context (Ha D3); C: soil control samples (m/z 191) from the Heuneburg, limit of the Plateau and the lower town settlement (HBSED06, Ha D3 layers), and D: absence of hopanes (m/z 191) in the soil linked to the external surface of a Plateau vessel (HB-PL-005, Ha D1).
(TIF)

S2 Fig Control sample from the lower town settlement context (absence of hopanes). GC-MS selected ion monitoring (m/z 191) of a handmade bowl (HB-VB-025) from the Heuneburg, lower town settlement context (Ha D1) showing A: the molecular signature of birch bark tar in the soil linked to the inner surface of the sherd, and B: the hopane distributions inside the sherd.
(TIF)

S3 Fig Control sample from the outer settlement context (absence of hopanes). GC-MS selected ion monitoring (m/z 191) of a handmade vessel (HB-AS-034) from the Heuneburg, outer settlement context (Ha D1) showing hopane distributions A: inside the sherd, and B: on the outer surface of the sherd.
(TIF)

S4 Fig Control sample from the Heuneburg soils (absence of tartaric acid). Chromatogram showing the molecular signature following BF3/BuOH treatment and extraction in DCM, [24] in A: Standards (including L-Malic and L-Tartaric acids,TMS-ether butyl-esters), B: inside a handmade bowl (HB-PL-019) from the Plateau, C: in the soil linked to the inner surface of a handmade bowl (HB-PL-019) from the Plateau, D: the soil control sample from limit of the Plateau and the low town settlement (HBSED06), and E: the soil linked to the inner surface of a handmade bowl (HB-VB-025) from the lower town settlement.
(TIF)

S5 Fig Organic substances identified in fine pottery from the Heuneburg of the Late Hallstatt period (unstratified contexts). (TIF)

S6 Fig Layer of birch bark tar applied to the bottom of a coarse vessel from the plateau (HB-PL-110; Photos B. Schorer). (TIF)
