# Supplementary figures and images for: The dynamics of Early Celtic consumption practices: A case study of the pottery from the Heuneburg
Source: PLoS One. 2019 Oct 23;14(10):e0222991. doi: 10.1371/journal.pone.0222991 (PMC6808335; doi:10.1371/journal.pone.0222991)

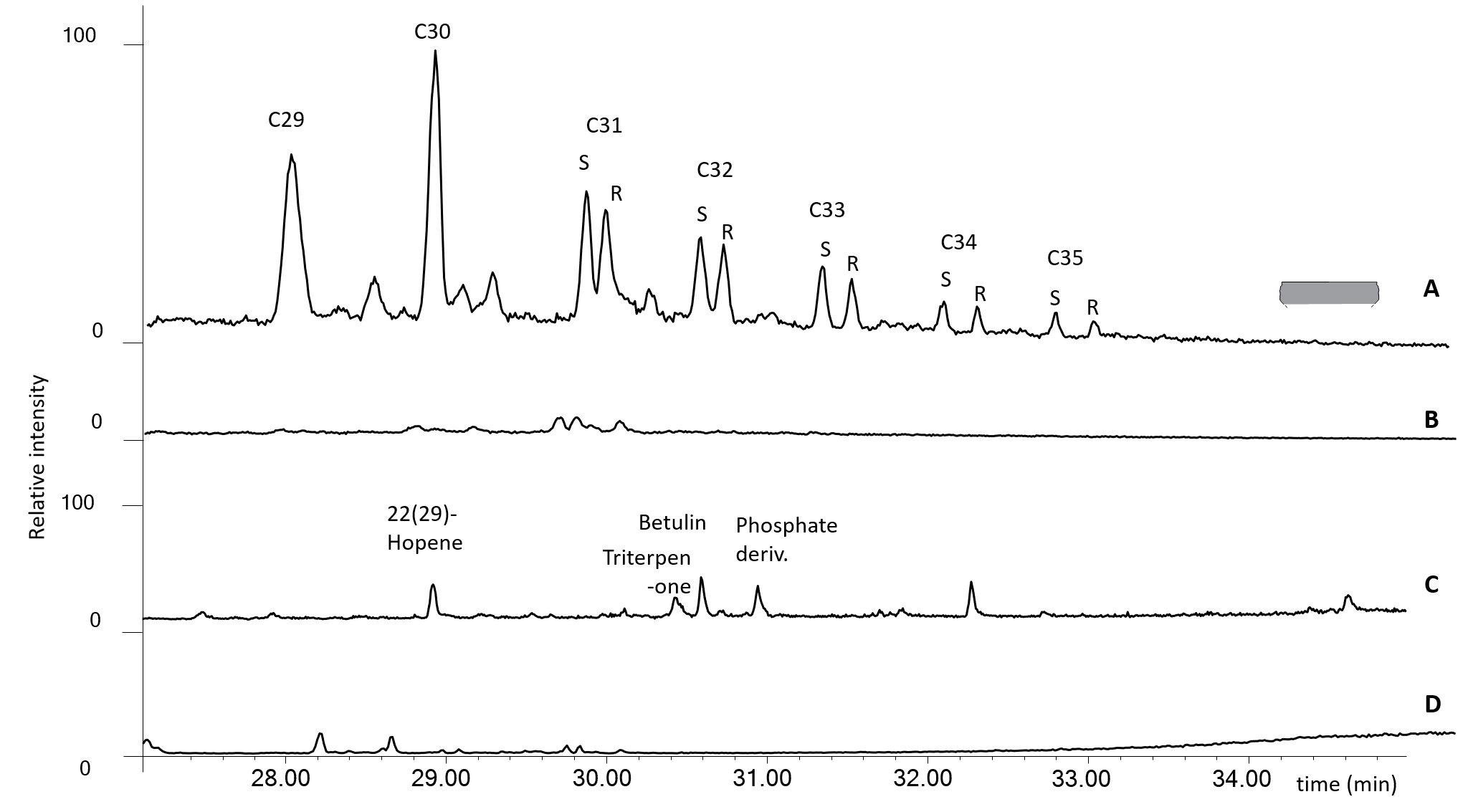

Supplement: S1 Fig — GC-MS selected ion monitoring showing: A: hopane distributions (m/z 191) inside wheel-made bowl (HBPL617) from the Heuneburg, Plateau context (Ha D3); B: absence of methylhopanes (m/z 205) inside wheel-made bowl (HBPL617) from the Heuneburg, Plateau context (Ha D3); C: soil control samples (m/z 191) from the Heuneburg, limit of the Plateau and the lower town settlement (HBSED06, Ha D3 layers), and D: absence of hopanes (m/z 191) in the soil linked to the external surface of a Plateau vessel (HB-PL-005, Ha D1). (TIF) [file pone.0222991.s002.tif]

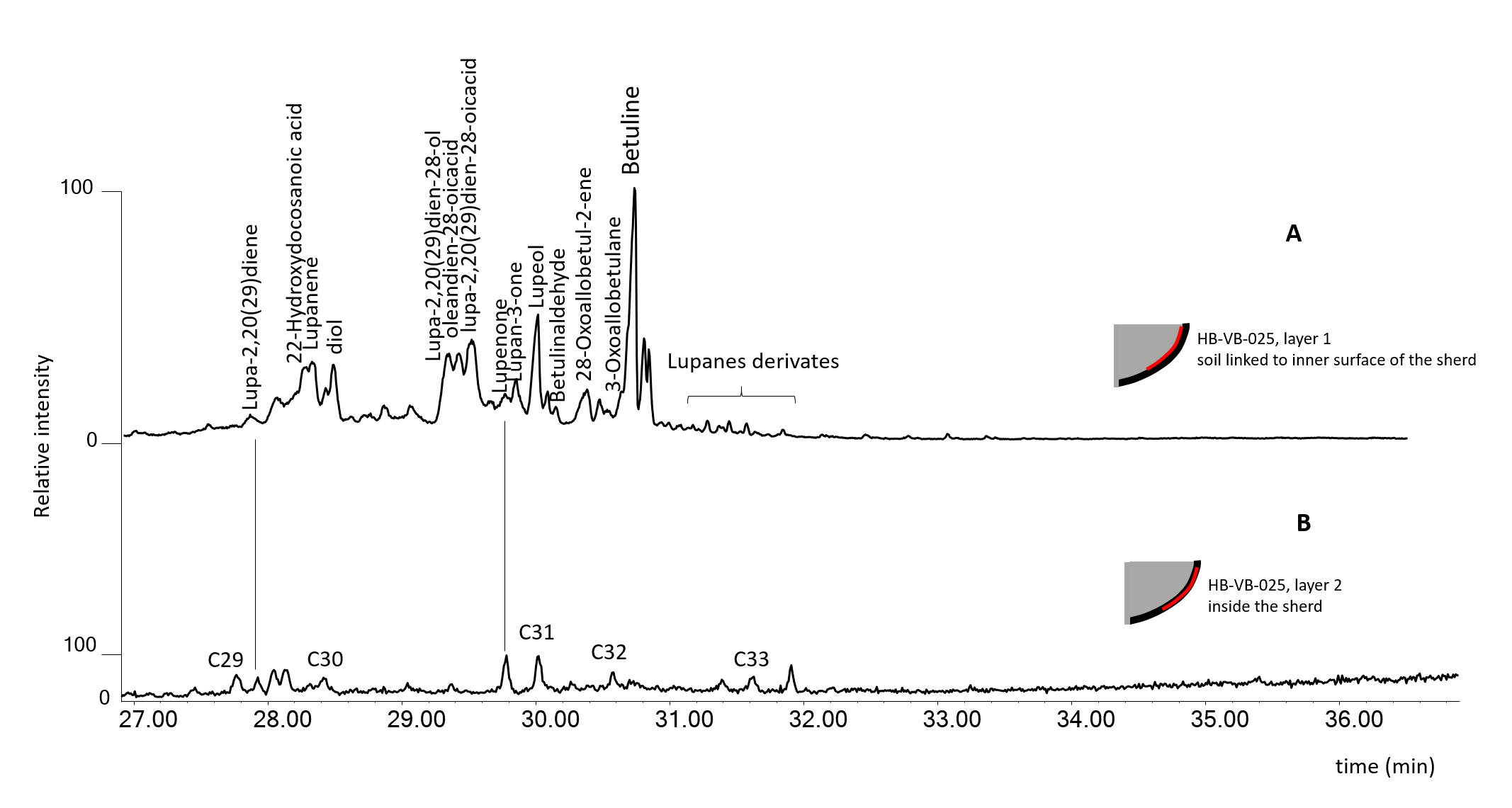

Supplement: S2 Fig — GC-MS selected ion monitoring (m/z 191) of a handmade bowl (HB-VB-025) from the Heuneburg, lower town settlement context (Ha D1) showing A: the molecular signature of birch bark tar in the soil linked to the inner surface of the sherd, and B: the hopane distributions inside the sherd. (TIF) [file pone.0222991.s003.tif]

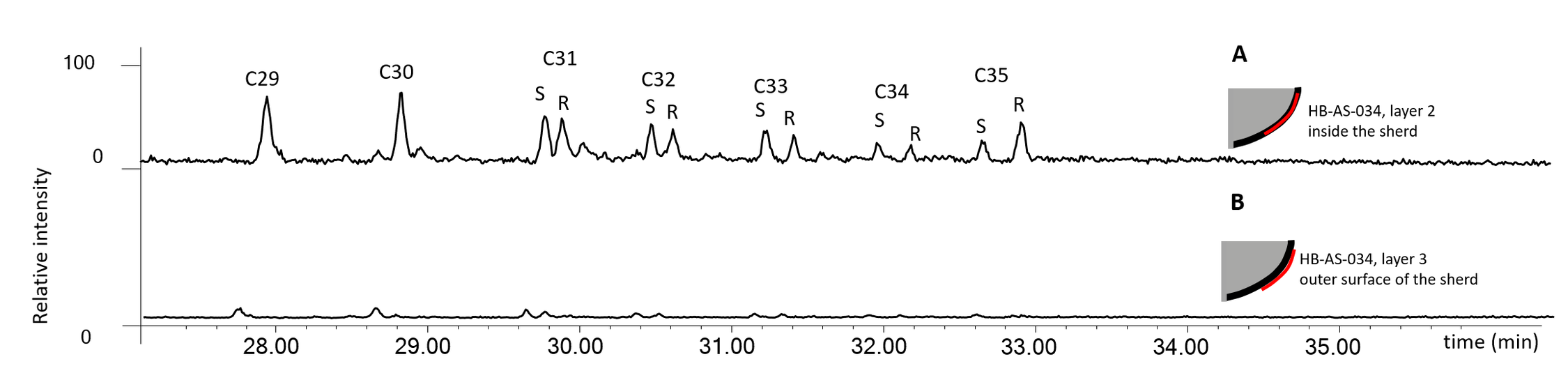

Supplement: S3 Fig — GC-MS selected ion monitoring (m/z 191) of a handmade vessel (HB-AS-034) from the Heuneburg, outer settlement context (Ha D1) showing hopane distributions A: inside the sherd, and B: on the outer surface of the sherd. (TIF) [file pone.0222991.s004.tif]

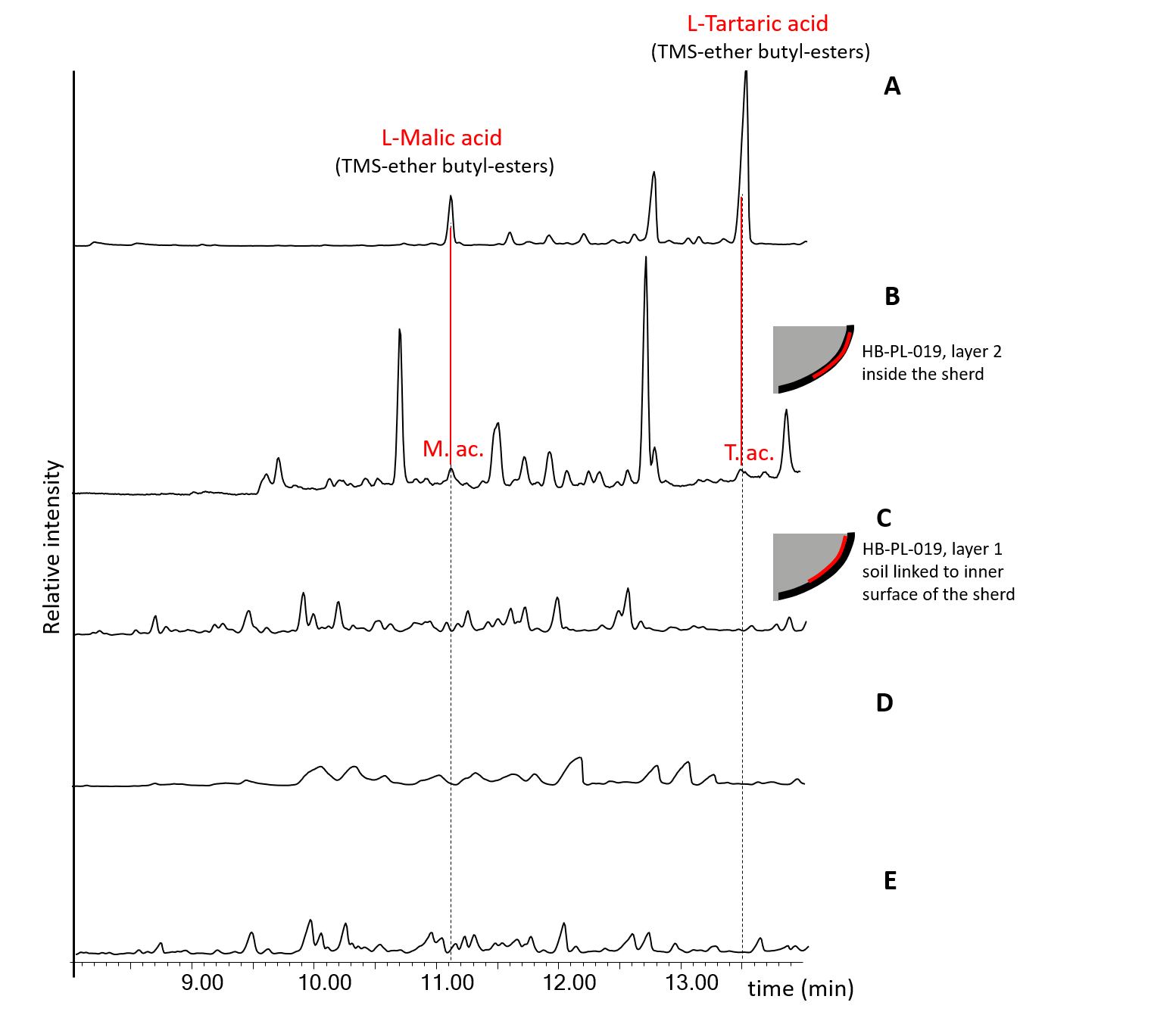

Supplement: S4 Fig — Chromatogram showing the molecular signature following BF3/BuOH treatment and extraction in DCM, [24] in A: Standards (including L-Malic and L-Tartaric acids,TMS-ether butyl-esters), B: inside a handmade bowl (HB-PL-019) from the Plateau, C: in the soil linked to the inner surface of a handmade bowl (HB-PL-019) from the Plateau, D: the soil control sample from limit of the Plateau and the low town settlement (HBSED06), and E: the soil linked to the inner surface of a handmade bowl (HB-VB-025) from the lower town settlement. (TIF) [file pone.0222991.s005.tif]

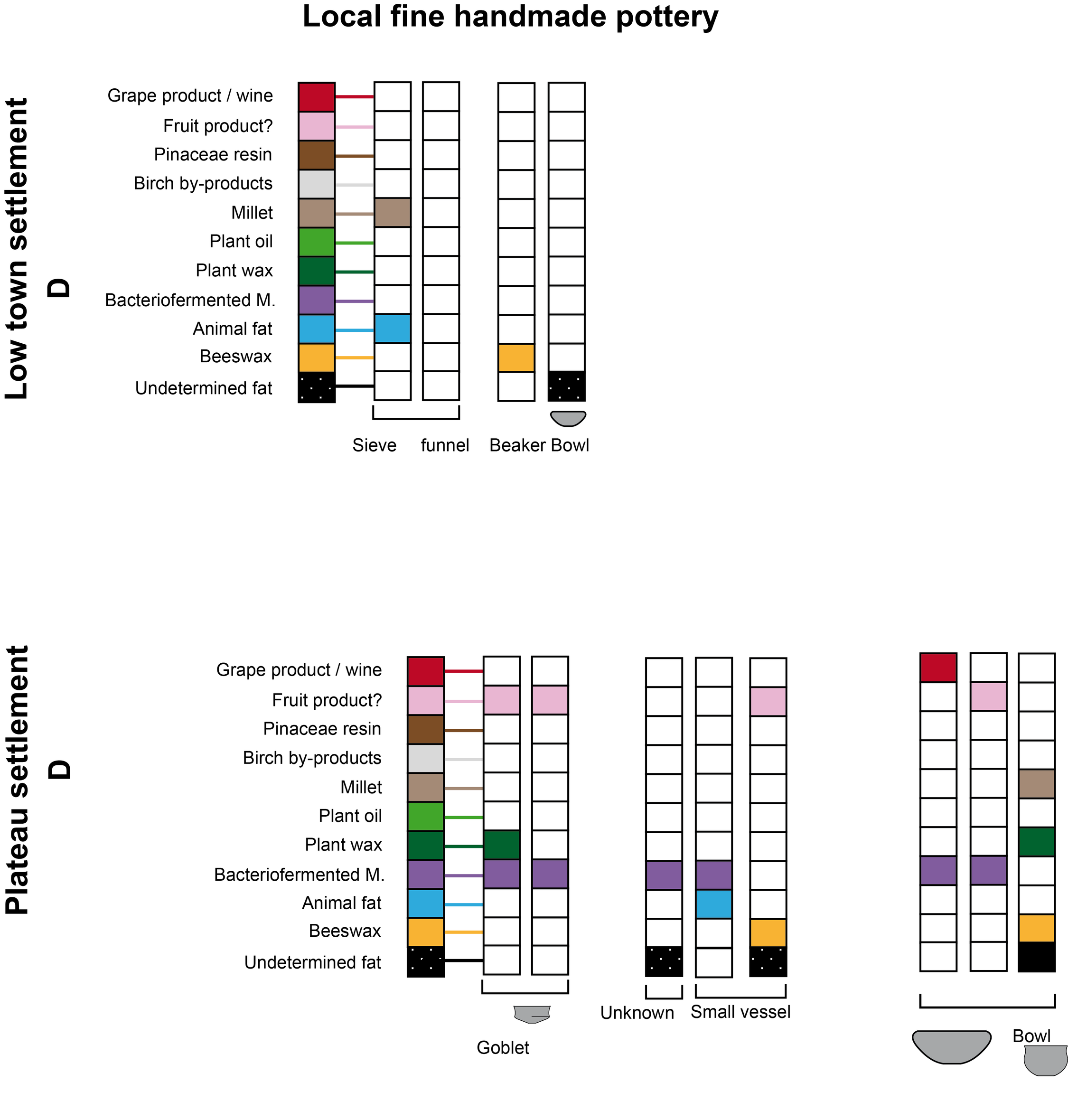

Supplement: S5 Fig — (TIF) [file pone.0222991.s006.tif]

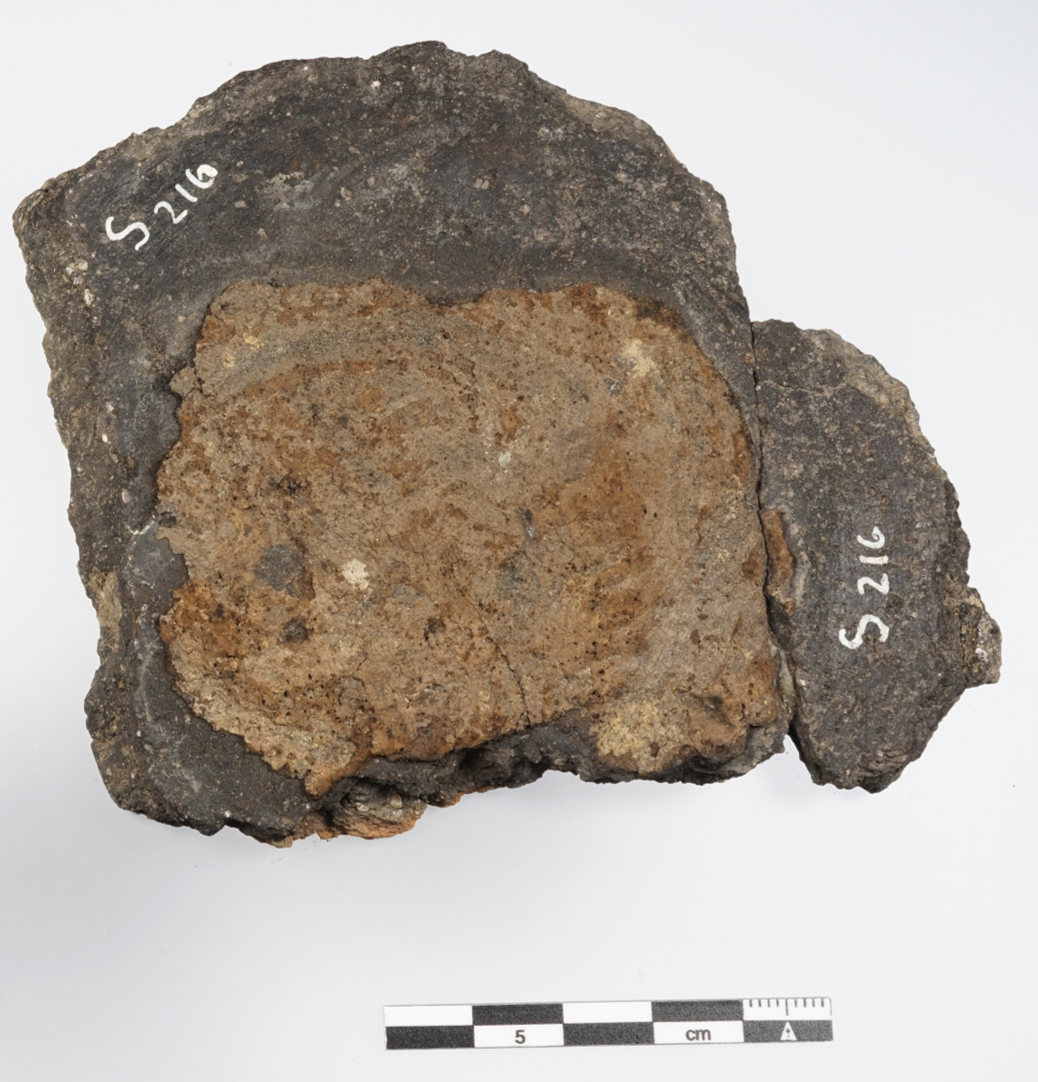

Supplement: S6 Fig — (TIF) [file pone.0222991.s007.tif]
